# Multivariate joint modeling to identify markers of growth and lung function decline that predict cystic fibrosis pulmonary exacerbation onset

**DOI:** 10.1186/s12890-020-1177-z

**Published:** 2020-05-19

**Authors:** E. R. Andrinopoulou, J. P. Clancy, R. D. Szczesniak

**Affiliations:** 1 Department of Biostatistics, Erasmus MC, Rotterdam, The Netherlands; 2 Cystic Fibrosis Foundation, Bethesda, MD USA; 3 Division of Pulmonary Medicine, Cincinnati Children’s Hospital Medical Center, Cincinnati, OH USA; 4 Department of Pediatrics, University of Cincinnati, Cincinnati, OH USA; 5 Division of Biostatistics & Epidemiology, Cincinnati Children’s Hospital Medical Center, Cincinnati, OH USA

**Keywords:** Dynamic prediction, Functional data analysis, Medical monitoring, Multivariate longitudinal data, Registry analysis, Time-to-event

## Abstract

**Background:**

Attenuated decreases in lung function can signal the onset of acute respiratory events known as pulmonary exacerbations (PEs) in children and adolescents with cystic fibrosis (CF). Univariate joint modeling facilitates dynamic risk prediction of PE onset and accounts for measurement error of the lung function marker. However, CF is a multi-system disease and the extent to which simultaneously modeling growth and nutrition markers improves PE predictive accuracy is unknown. Furthermore, it is unclear which routinely collected clinical indicators of growth and nutrition in early life predict PE onset in CF.

**Methods:**

Using a longitudinal cohort of 17,100 patients aged 6–20 years (US Cystic Fibrosis Foundation Patient Registry; 2003–2015), we fit a univariate joint model of lung-function decline and PE onset and contrasted its predictive performance with a class of multivariate joint models that included combinations of growth markers as additional submodels. Outcomes were longitudinal lung function (forced expiratory volume in 1 s of % predicted), percentiles of body mass index, weight-for-age and height-for-age and PE onset. Relevant demographic/clinical covariates were included in submodels. We implemented a univariate joint model of lung function and time-to-PE and four multivariate joint models including growth outcomes.

**Results:**

All five joint models showed that declining lung function corresponded to slightly increased risk of PE onset (hazard ratio from univariate joint model: 0.97, *P* < 0.0001), and all had reasonable predictive accuracy (cross-validated area under the receiver-operator characteristic curve > 0.70). None of the growth markers alongside lung function as outcomes in multivariate joint modeling appeared to have an association with hazard of PE. Jointly modeling only lung function and PE onset yielded the most accurate (area under the receiver-operator characteristic curve = 0.75) and precise (narrowest interquartile range) predictions. Dynamic predictions were accurate across forecast horizons (0.5, 1 and 2 years) and precision improved with age.

**Conclusions:**

Including growth markers via multivariate joint models did not yield gains in prediction performance, compared to a univariate joint model with lung function. Individualized dynamic predictions from joint modeling could enhance physician monitoring of CF disease progression by providing PE risk assessment over a patient’s clinical course.

## Background

**Fig. 1:**
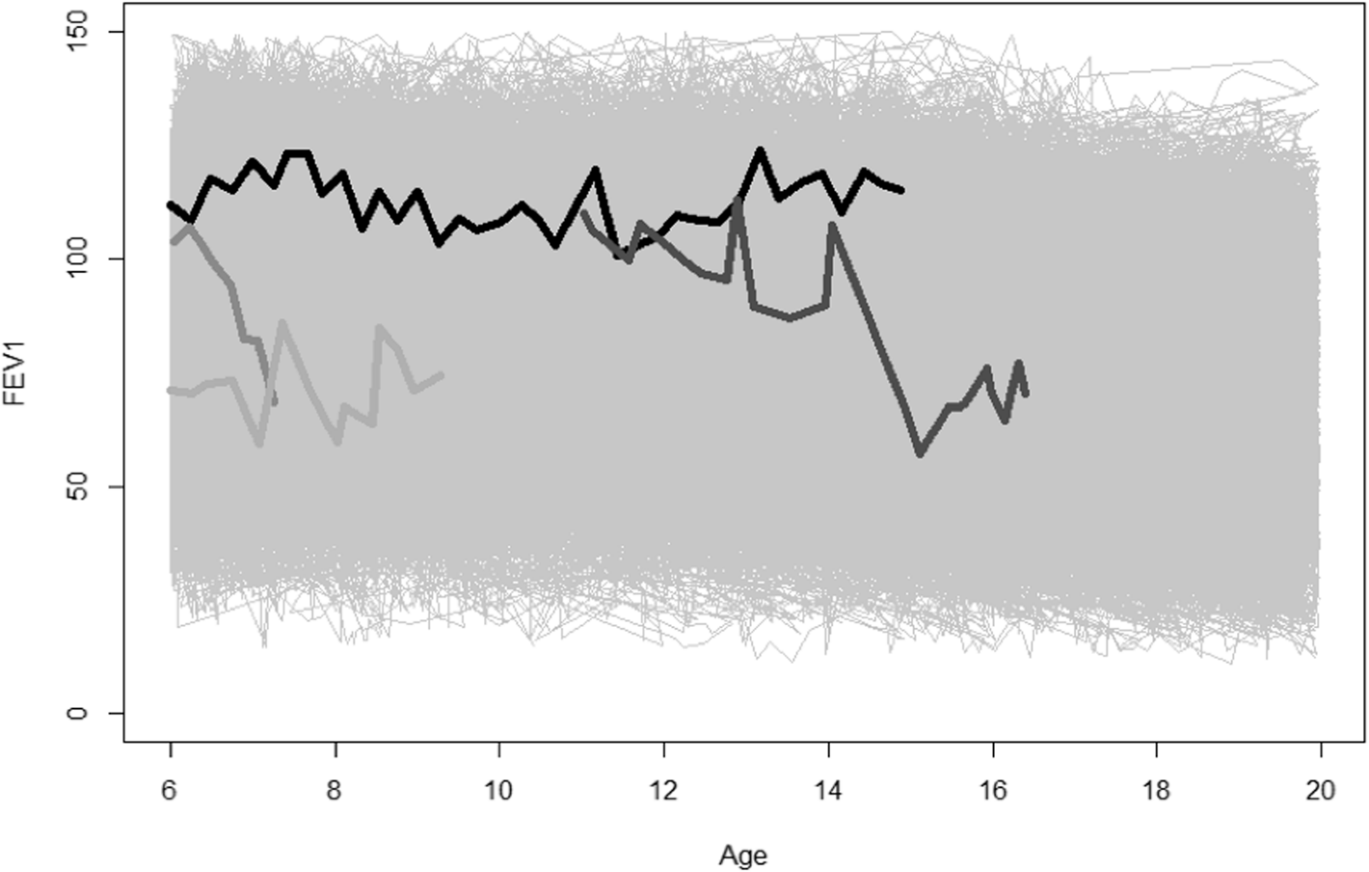
Lung function profiles over time. FEV1 (% predicted on the y-axis) is shown against time (age, in years, on the x-axis). Four representative patients are shown with thicker and darker-shaded trajectories, while remaining patients’ trajectories are thinner and in light gray. Two of the selected profiles (which appear lower overall, compared to the other two profiles) correspond to two individuals who experienced pulmonary exacerbations

Cystic fibrosis (CF) is a chronic lung disease in which death commonly results from respiratory failure [1]. A cycle of prolonged and acute drops in lung function marks the clinical course of CF [2], most notably during adolescence and early adulthood [3–5]. Forced expiratory volume in 1 s of % predicted (hereafter, FEV_1_), is the primary surrogate of lung function and strongest predictor of mortality in the CF population [6]. FEV_1_ remains an important outcome in clinical trials and the most relevant clinical indicator in monitoring lung function decline [7]. Fitting longitudinal FEV_1_ trajectories is critical to understanding disease progression, but it can be difficult to accurately depict the substantial variation that this marker exhibits both between patients and within an individual patient over time (Fig. 1). It is also of clinical importance to identify an acute respiratory event referred to as a pulmonary exacerbation (PE), which frequently corresponds to sharp decreases in lung function. Diagnosis of a PE depends on various factors corresponding to lung function and nutritional status, often resulting in a patient being hospitalized and administered intravenous antibiotics [8]. Further, patients who experience a PE often fail to recover to their corresponding pre-event (i.e., “baseline”) levels of FEV_1_ [9]. For these reasons, it has been desirable in epidemiologic studies to identify risk factors for having a PE.

Although maintaining lung function and minimizing risk of PEs are essential to survival, CF is a multi-system disease in which malnutrition and poor growth have been shown to adversely impact pulmonary function [10]. Body mass index percentile (BMIp) is the primary nutritional indicator in CF [11]; guidelines published by the CF Foundation recommend maintaining BMIp >50th percentile [12]. A recent CF registry analysis found that weight-for-age (WFA) and height-for-age (HFA) percentiles more accurately identify CF patients of poor nutritional status and stunting, respectively, compared to BMI percentiles [13].

These and other studies have collectively demonstrated that multiple causes of disease progression are typically observed from indicators of pulmonary and nutritional decline, yet it remains unclear how the underlying longitudinal processes collectively evolve over time. Joint modeling is a popular statistical approach used to link longitudinal and time-to-event processes and is usually implemented through shared parameters [14]. These models assume that the longitudinal and event processes are correlated through random effects [15]. In particular, we describe the evolution over time for the longitudinal outcomes with a mixed-effects model. Then, we use these estimated evolutions as time-varying covariates in the survival model.

Novel applications of so-called univariate joint models (refers to a single longitudinal marker being modeled simultaneously with an event outcome) in CF epidemiologic studies have yielded new insights and predictions regarding how changes in FEV_1_ are associated with survival (i.e., time to death/lung transplant) [16–18]. Although these applications elucidate CF disease progression into its end stage, little is known about the utility of joint models in assessing onset of PEs; these events initialize earlier in life but could be attributable to downstream impacts. Furthermore, to our knowledge, the association of one or more longitudinal outcomes with onset of PE has not yet been addressed. Univariate joint modeling potentially ignores the fact that different markers of interest, specifically those related to growth and nutrition status, could also have an influence on PE in combination.

Due to computational advancements, such as new software packages [19], joint models have enjoyed a renaissance in the last decade [20]. In this paper, we focus on multiple longitudinal outcomes and their potential to predict PE onset in children with CF. Specifically, we propose a multivariate joint model of FEV_1_ and markers of nutritional status with time-to-PE. The objective of our study was to construct these models to estimate how commonly collected markers of these longitudinal processes evolve through the clinical course of CF, and to compare results from these models regarding predictive accuracy. We utilize data from the CFF patient registry and provide implementation of the joint models as supplemental material.

## Methods

### Cohort description

The CF cohort for this empirical study consists of patients with longitudinal data recorded from childhood until early adulthood (ages: 6–20 years) in the US Cystic Fibrosis Foundation Patient Registry (CFFPR) between January 1, 2003, and December 31, 2015. Patients younger than 6 years of age were excluded due to potentially unreliable pulmonary function testing. To focus the study on the most modern era of CF care, we considered available data from 2003 onward. A detailed description of this registry and its contents is provided elsewhere [21].

### Outcomes

To build the multivariate joint model for this cohort, we focused on established outcomes of lung function and nutrition. Outcomes on nutrition and growth, available from the CFFPR and computed based on formulas from the Centers for Disease Control and Prevention, included the aforementioned percentiles for BMIp, WFA and HFA. Observed FEV_1_ was expressed as percentage of predicted using established reference equations for age, sex and race [22, 23]. Occurrence of first PE, the event outcome, was subject to censoring and considered to have occurred if documented in the CFFPR as warranting treatment with intravenous antibiotics in the hospital.

### Covariates

Covariates from the aforementioned literature were considered, as well as findings from analyses specified in the most recent CFFPR report. These included genotype (F508del homozygous, heterozygous or neither/unknown), sex, Hispanic ethnicity, socioeconomic status (SES, having only state/federal or no insurance was recorded as 1, and 0 otherwise); time-varying covariates included use of pancreatic enzymes (corresponding to pancreatic insufficiency), CF-related diabetes (CFRD, with or without fasting hyperglycemia), positive cultures for *methicillin-resistant Staphylococcus aureus* (*MRSA*) and *Pseudomonas aeruginosa* (*Pa*).

Baseline was defined as the time at which all longitudinal outcomes were first recorded during the eligibility period. A birth cohort variable was used to account for potential delayed entry into the registry and changes due to advancements in CF care and therapeutics approvals. Another time-varying covariate was used to account for irregular sampling bias, potentially induced by patients having a varying number of clinical encounters over time; for a given patient and encounter, this variable was the total number of encounters within the prior year. Data acquired after lung transplant were excluded.

### Descriptive analysis

Descriptive statistics, including median (Q1-Q3) for continuous variables and n (%) for categorical variables, were used to summarize cohort characteristics and extent of follow-up available for each patient.

### Joint model setup and notation

Prior to multivariate joint modeling, we performed a univariate joint model that included only FEV_1_ and time-to-first PE. To model the association between combinations of longitudinal FEV_1_ and other outcomes together with time-to-first PE, we examined multivariate joint models. We describe the notation using a particular multivariate joint model with two longitudinal outcomes, which can be generalized to the case of three outcomes (e.g. FEV_1_, WFA and HFA). We assume *y*_*1i*_*(t)* and *y*_*2i*_*(t)* to be the follow-up measurements (e.g., FEV_1_ and BMIp, respectively) for patient *i (i = 1, …, n)* at a specific time point *t*. We follow the framework of mixed-effects models to model the longitudinal outcomes:

$$ {y}_{1i}\left(\mathrm{t}\right)={x}_{1i}^T(t){\beta}_1+{z}_{1i}^T(t){b}_{1i}+{\varepsilon}_{1i}(t)={m}_{1i}(t)+{\varepsilon}_{1i}(t) $$

and

$$ {y}_{2i}\left(\mathrm{t}\right)={x}_{2i}^T(t){\beta}_2+{z}_{2i}^T(t){b}_{2i}+{\varepsilon}_{2i}(t)={m}_{2i}(t)+{\varepsilon}_{2i}(t), $$

where ${\mathrm{x}}_{1\mathrm{i}}^{\mathrm{T}}\left(\mathrm{t}\right)$ and ${\mathrm{x}}_{2\mathrm{i}}^{\mathrm{T}}\left(\mathrm{t}\right)$ denote the design vectors for the fixed effects regression coefficients β_1_ and β_2_. Moreover, ${\mathrm{z}}_{1\mathrm{i}}^{\mathrm{T}}\left(\mathrm{t}\right)$ and ${\mathrm{z}}_{2\mathrm{i}}^{\mathrm{T}}\left(\mathrm{t}\right)$ denote the design vectors for the random effects b_2i_ and b_1i_. We included natural cubic splines to accommodate nonlinear progression of different covariate levels over age with respect to each longitudinal outcome, using an approach similar to our previous work in modeling nonlinear age-related FEV_1_ [5]) [24]). Furthermore, the longitudinal outcomes FEV_1_ and BMIp are correlated through the random effects b_i_ = {b_1i_, b_2i_}, where a multivariate normal distribution is assumed.

For the PE part, we assume *T*_*i*_ to be the observed failure time for patient *i*, as ${T}_i=\min \left({T}_i^{\ast },{C}_i\right)$ where ${T}_i^{\ast }$ indicates the true failure time of individual *i*^*th*^ experiencing the event (occurrence of PE) and *C*_*i*_ the censored time. We assume a proportional hazard model for the risk of the event:

$$ \text{h}_{\text{i}}(\text{t}) = \text{h}_{0}(\text{t}) \text{exp}\{{\omega}^{\text{T}}_{\text{i}}\gamma + \text{a}_{1}\text{m}_{1\text{i}}(\text{t})+ \text{a}_{2}\text{m}_{2\text{i}}(\text{t})\}, $$

where ${\omega}_i^T$ denotes row vectors of the design matrix of the baseline covariates ,*γ* is the corresponding regression coefficients vector, and *a*_1_ and *a*_2_ are the coefficients that connect the longitudinal and event processes. In our case, these parameters represent the strength of the association between FEV_1_ and BMIp with onset of PE. To better understand the connection between the event and longitudinal parts in our model, we explain the meaning of the *a*_1_ and *a*_2_ parameters. Specifically, for a one unit increase in the underlying value of FEV_1_ for patient *i*, the hazard ratio is exp(a_1_) assuming that the baseline covariates and BMIp remain the same. Similarly, exp(a_2_) is the hazard ratio when the underlying value of BMIp for the *i* -th patient is increased by one unit assuming that the baseline covariates and FEV_1_ remain the same.

### Implementation

We employed Markov-Chain Monte-Carlo (MCMC) via Gibbs sampling using the ‘JMbayes’ package (Version 0.8–82) in R (Version 3.5.3., R Foundation for Statistical Computing, Vienna Austria) to obtain the parameters from the respective posterior distributions under the multivariate joint model [19]. The highest posterior density (HPD) and accompanying standard errors were used to estimate each parameter of interest. Due to the large patient sample size that led to memory problems, we randomly divided the dataset into three parts (each part had similar percentage of PE at onset). Each of the datasets was fitted separately, and for the final results we combined the MCMC samples as described previously [25]. Code implementation is provided as supplemental material. In total, we fit five joint models wherein PE onset was the event: i) only FEV_1_; others included ii) BMIp; iii) HFA; iv) WFA; v) WFA and HFA. The multivariate joint models in (ii) – (iv) were implemented to evaluate the impact of different measures of nutritional status on predicting PE onset. Due to the large number of parameters in scenario (v), we assumed a less flexible evolution for FEV_1_ and BMIp over time. In particular, we postulate natural cubic splines with two degrees of freedom to estimate this evolution instead of natural cubic splines with three degrees of freedom assumed in the other scenarios.

### Evaluating predictive performance

We calculated the area under the receiver-operator characteristic curve (AUC) to evaluate predictive accuracy of each model with respect to PE events using ten-fold cross validation. A large value of AUC indicates the preferred model. To obtain correct estimates of the AUC we performed a 5-fold cross validation procedure, wherein each time we selected 700 individuals from the large data set and we split it in five subsets. Each time we fitted the model in four of the subsets, we calculated the AUC in the subset that was excluded. The calculation of the AUC was performed at 12 and 16 years of age with prediction windows of 0.5, 1, 2 years, in order to mimic clinically meaningful age strata and prediction windows. This procedure was repeated 100 times. Appropriate diagnostics for joint models were examined. The code of the analysis and cross validation are shown in the Supplement.

## Results

### Analysis cohort

**Fig. 2:**
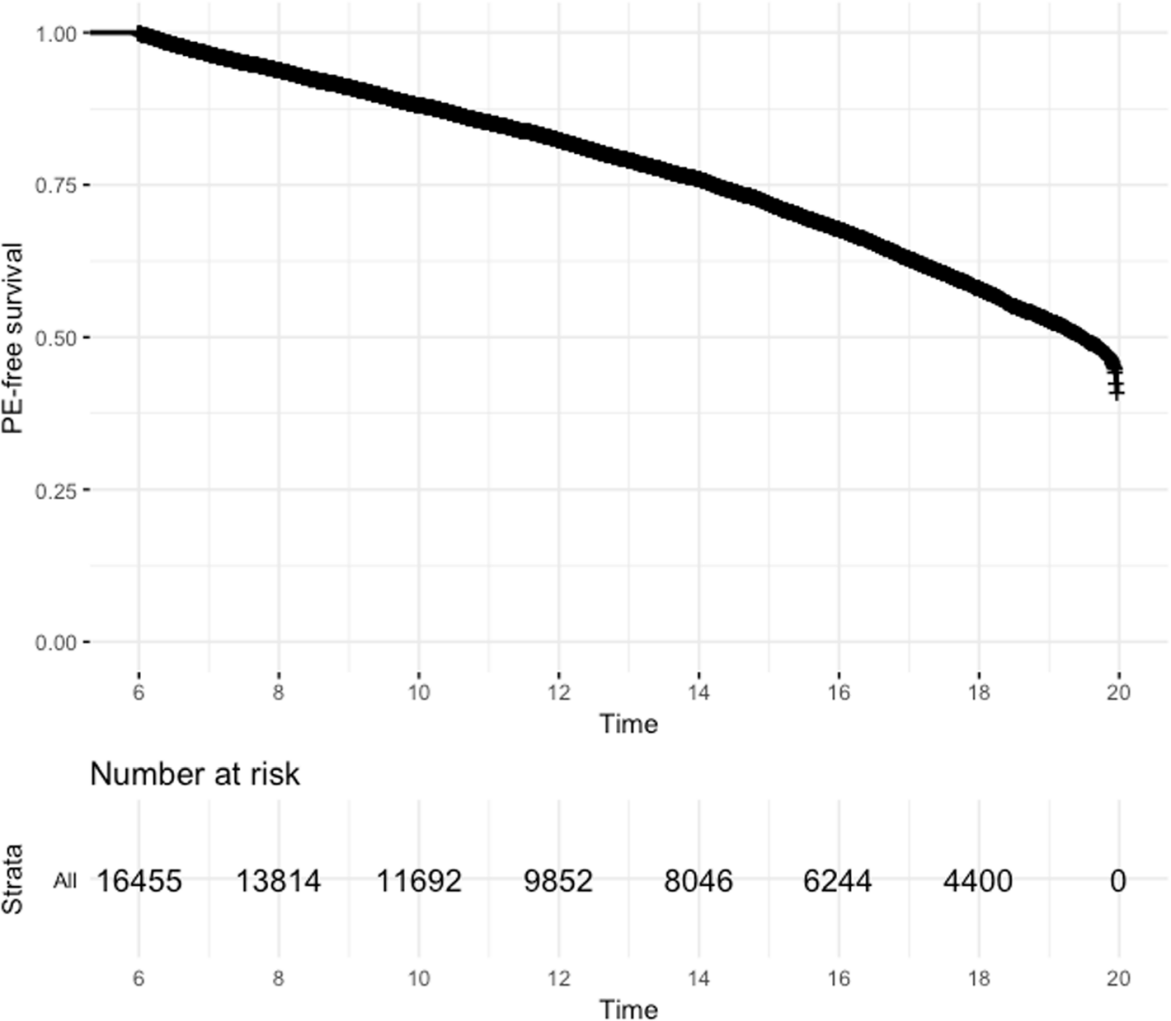
Kaplan-Meier plot of time to first recorded pulmonary exacerbation. Time is expressed as age (in years on the x-axis) and PE-free survival probability (y-axis) corresponds to probability of not experiencing a pulmonary exacerbation. Data shown include all patients

There were 16,455 patients contributing 245,513 observations to the analysis cohort (Table 1). Patients who had a PE during follow-up tended to be female and had the F508del homozygous genotype. They had slightly higher proportions of infections with MRSA and Pa than patients who did not have a PE during follow up. By contrast, patients who were PE-free during follow-up had a higher reported use of pancreatic enzymes and had higher levels of lung function and better nutrition status at baseline. Extent of follow-up also differed according to PE onset. Representative trends of lung function decline according to PE status are shown in Fig. 1 and reflect summary results from Table 1. Median (95% CI) age of PE onset was 19.5 (19.3–19.6) years (Fig. 2).

**Table 1:** Cohort characteristics according to PE onset*

Characteristic at Baseline	Had PE during follow up (*n* = 5510)	PE-free during follow up (*n* = 10,945)	Overall (*n* = 16,455)
Genotype^			
Homozygous	53.0%	45.6%	48.1%
Heterozygous	36.4%	39.1%	38.2%
None/unknown	10.6%	15.3%	13.7%
Male sex^	47.4%	53.7%	51.6%
Hispanic	9.2%	8.5%	8.7%
Birth cohort^			
< 1981	16.7%	19.8%	18.8%
1981–1989	31.6%	22.2%	25.3%
1990–1994	22.0%	13.9%	16.6%
1995–1999	24.6%	26.9%	26.1%
> 1999	5.1%	17.2%	13.2%
Age at entry, years^	8.7 (6.2–13.0)	7.9 (6.2–14.0)	8.2 (6.2–13.6)
Low SES	54.0%	52.4%	49.8%
MRSA^	9.5%	6.4%	7.4%
Pa^	24.2%	17.3%	19.6%
CFRD^	5.1%	3.2%	3.9%
Pancreatic insufficient^	32.3%	42.3%	39.0%
FEV_1_, % predicted^	83.8 (67.3–98.0)	93.5 (79.3–105.2)	90.6 (74.9–103.2)
BMIp, percentile^	43.1 (19.9–67.0)	51.1 (27.3–73.4)	48.6 (24.8–71.3)
WFA, percentile^	29.1 (10.2–55.0)	38.9 (16.7–64.7)	35.5 (14.3–61.7)
HFA, percentile^	24.5 (8.4–49.8)	32.4 (12.3–59.5)	29.7 (10.7–56.7)
Length of follow up, years^	1.9 (0.5–4.4)	3.1 (1.0–6.3)	2.6 (0.8–5.7)
Alive^	99.9%	99.7%	99.8%

Abbreviations: *BMIp* body mass index percentile, *CFRD* cystic fibrosis related diabetes, *FEV*_*1*_ forced expiratory volume in 1 s, *HFA* height for age, *MRSA* methicillin-resistant *Staphylococcus aureus*, *Pa Pseudomonas aeruginosa*, *PE* pulmonary exacerbation, *SES* socioeconomic status, *WFA* weight for age. * Values at first recorded entry in the database are used for all patients except for follow-up and death; results for continuous and categorical variables are expressed as median (Q1-Q3) and % over column total, respectively. Characteristics marked as ^ imply *P* < 0.05 for comparison between PE and PE-free groups. Between-group frequencies compared using Chi-square test of independence; continuous variables compared between groups using Welch two-sample t-test

### Joint model estimates

Each Gibbs sampler ran for 900 iterations, and the first 600 iterations were discarded for burn-in. Shared parameter estimates, which represent associations between each longitudinal process and the PE event, are shown for all five fitted joint models (Table 2). The log-hazard estimates of the shared parameters, which were introduced in Section 3.2, indicate that FEV_1_ was consistently and negatively associated with PE onset. Corresponding hazard ratio estimates ranged from 0.96 to 0.97, suggesting a small association between changes in the FEV_1_ trajectory and PE onset. Jointly modeling BMIp, WFA or HFA as shown in respective models (ii) – (iv) did not impact association between FEV_1_ and PE onset, and their associations with PE onset did not reach statistical significance.

**Table 2:** Associations between longitudinal processes and risk of PE*

	log (HR) estimates (SE) and *P*-value			
Joint Model	FEV_1_	BMIp	WFA	HFA
I – Null	−0.0305 (5e-04) *P* < 0.001	–	–	–
II – BMIp	−0.0324 (6–04) *P* = 0.002	− 0.0004 (3–04) *P* = 0.988	–	–
III – WFA	−0.0334 (7e-04) *P* = 0.003	–	0.0006 (2e-04) *P* = 0.761	–
IV – HFA	−0.0308 (5e-04) *P* = 0.001	–	–	0.0004 (1e-04) *P* = 0.927
V – WFA and HFA	−0.0327 (6 e-04) *P* = 0.005	–	0.0022 (9e-04) *P* = 0.683	−0.0019 (1e-03) *P =* 0.737

Abbreviations: *BMIp* body mass index percentile, *FEV*_*1*_ forced expiratory volume in 1 s, *HFA* height-for-age percentile, *HR* hazard ratio, *PE* pulmonary exacerbation, *WFA* weight-for-age percentile. *Associations estimated as log hazard using the posterior mean (SE) with corresponding *p*-value in the Bayesian sense. Log-hazard estimates > 0 (< 0) imply positive (negative) association between the longitudinal process and PE onset. Results are reported as HRs in text. Each model includes all covariates (see Methods). All joint model parameter estimates are provided in Supplement S1

The univariate joint model (i) in Table S1 indicated that being Hispanic and having lower SES, MRSA and CFRD and using pancreatic enzymes corresponded to worse overall FEV_1_. There were nonlinear associations, as reflected by spline coefficients, between each of genotype, sex, SES, MRSA, Pa and CFRD and FEV_1_ decline. In the PE event submodel, patients who were male, used pancreatic enzymes, had lower SES, infection with MRSA and Pa and were diagnosed with CFRD had greater risk of PE onset. The multivariate joint models, each of which included a longitudinal submodel for FEV_1_, had similar association results for the clinical/demographic covariates and shared parameters regarding FEV_1_.

The multivariate joint model (ii) in Table S2, which included a longitudinal submodel for BMIp, showed that being born into an older birth cohort, taking pancreatic enzymes and having more frequent clinic visits corresponded to higher BMIp. There were nonlinear associations between each of genotype, SES, birth cohort and Pa with BMIp trajectory. In Table S3, using height in multivariate joint model (iii), we found that having a F508del homozygous genotype and belonging to an older birth cohort were positively associated with overall HFA. By contrast to the BMIp submodel, having more frequent clinic visits corresponded to lower HFA. Similar nonlinear associations were observed in the BMIp and HFA submodels. Results for the WFA submodel in (iv) were also similar (Table S4). The multivariate joint model in (v), compared to models (iii) and (iv), had slight changes in associations shown in the WFA submodel; however, the HFA submodels were similar.

### Predictive performance

**Fig. 3:**
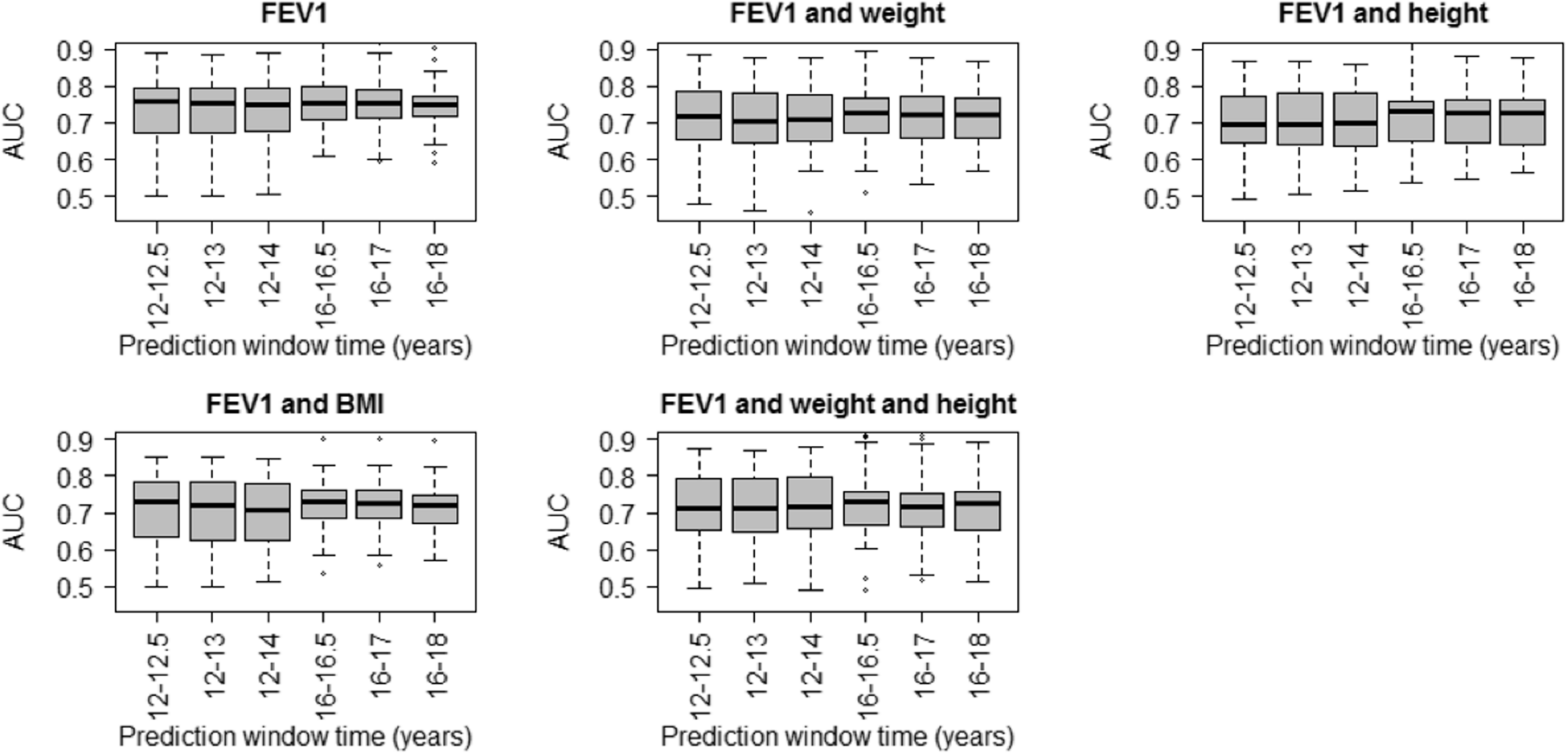
Cross-validation metrics from joint model. Results summarized as box plots show ability of each joint model to predict pulmonary exacerbation onset as estimated by area under the curve (AUC, y-axis)**,** stratified by age and forecast horizons (x-axis)

The results of the predictive performance of the joint models presented in Table 2 are summarized in Fig. 3. In particular, the AUC of each model is presented assuming a different prediction interval (0.5, 1 and 2 years), which starts at adolescence or early adulthood (ages 12 and 16 years, respectively). The median AUC values ranged from 0.7 to 0.8. Moreover, the univariate joint model, assuming only FEV_1_, provided the highest AUC value and was most robust across age strata and prediction windows. Precision of the predictions was assessed by examining the interquartile ranges of the boxplots in Fig. 3. Precision was lower for predictions done at adolescence, regardless of the interval, compared to precision estimated during young adulthood. Precision was highest under the univariate joint model for predicting PE risk at age 16 years out to 2 years. In terms of overall precision, the multivariate joint models performed similarly well.

**Fig. 4:**
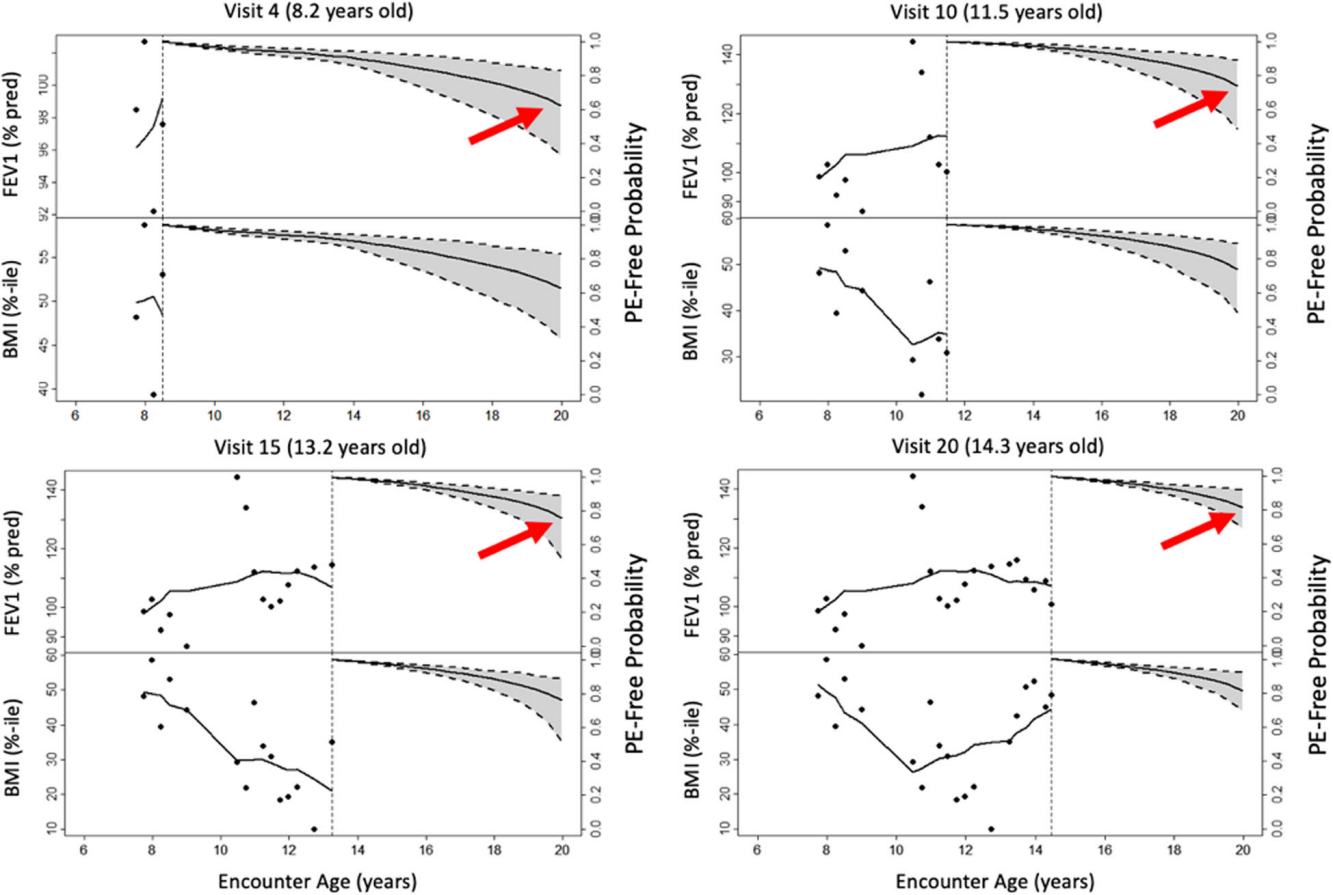
Observed and predicted evolution of markers of lung function and growth. Lung function (FEV1% predicted, upper left y-axis) and growth (BMIp, lower left y-axis) and probability of not having a pulmonary exacerbation (PE-free probability, upper/lower right y-axis) in a female CF F508del homozygous patient over a series of four clinical encounters (observations are black dots; mean response is black curve). Arrows illustrate how her probability of remaining PE-free is dynamically updated with each clinical encounter

**Fig. 5:**
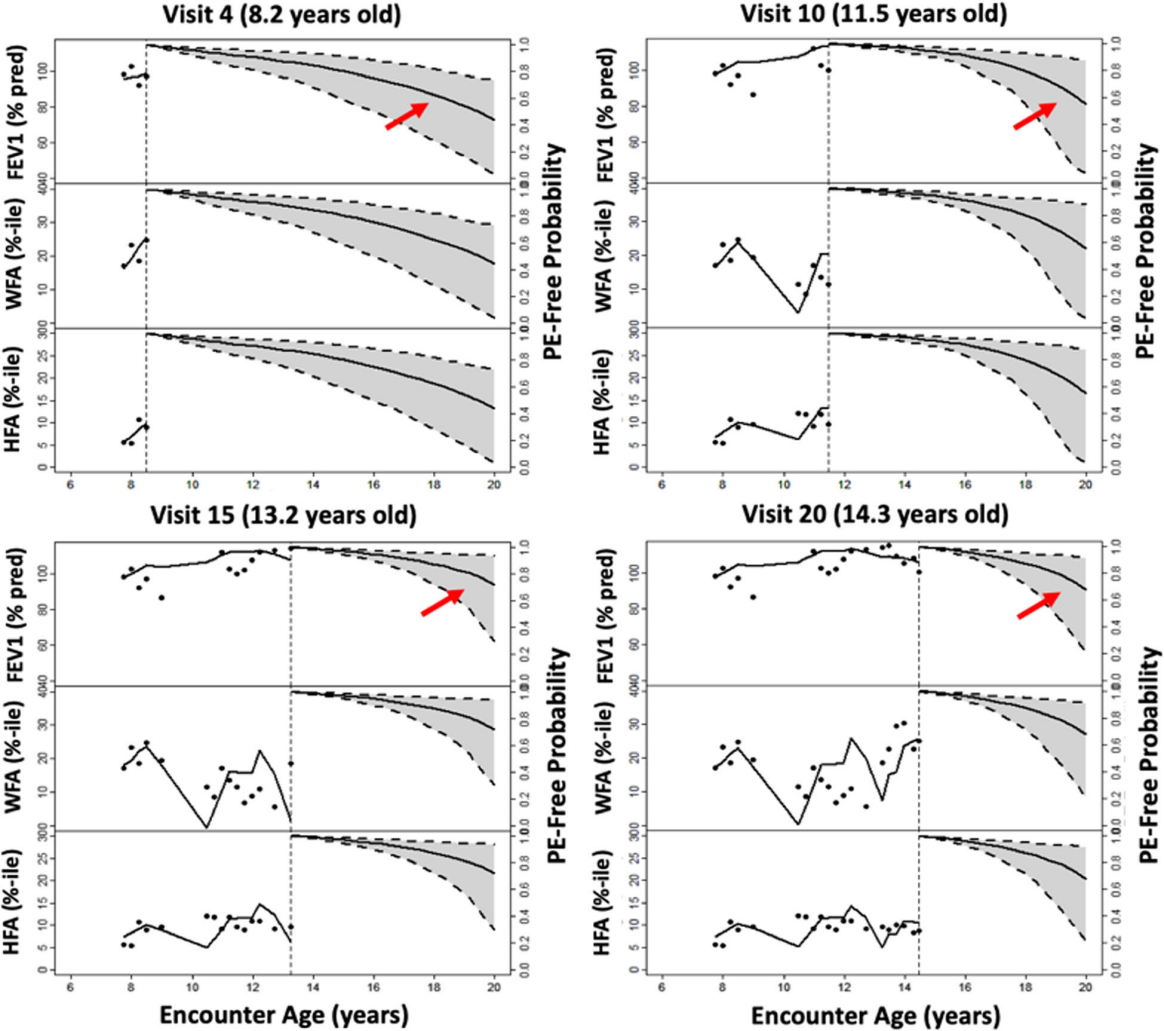
Multivariate joint model predictions with other growth markers. Same female CF patient as in Fig. 4, but simultaneously modeling lung function (FEV1, upper-left y-axis), growth as weight-for-age (WFA, middle-left y-axis) height-for-age (HFA, lower-left y-axis) percentiles and probability of not having a pulmonary exacerbation (PE-free probability, repeated along the right y-axis). Her probability of remaining PE-free is similar to Fig. 4 but estimates are less precise based on 95% CI over the four clinical encounters

Dynamic predictions for an individual CF F508del homozygous patient (born 1990–1994) are shown in Fig. 4 for the multivariate joint model of longitudinal FEV_1_ and BMIp. Her first recorded FEV_1_ and BMIp were 98.5% predicted and at the 48.2 percentile, respectively. She began taking pancreatic enzymes and had a positive culture for Pa around 10 years old. Her monitored outcomes are depicted across four clinic visits from ages 8.2 to 14.3 years, along with probability of not having a PE over follow up. Her long-term risk of PE appears higher during an earlier visit (e.g., PE-free probability is 0.6), compared to later visits that are informed by dynamic predictions from the multivariate joint models (e.g., PE-free probability increased to 0.8). Commensurate with these results are the changes in her FEV_1_ and BMIp trajectories, which imply minimal rate of decline and improving nutritional status, respectively. She did not experience a PE event during follow up. Her projected PE-free probabilities over follow up using a multivariate joint model replacing BMIp with WFA and HFA were similar (Fig. 5); however, precision with which PE-free probability could be estimated was decreased using this combination of markers rather than BMIp.

## Discussion

Past epidemiologic studies of PE onset have largely focused on explanation of risk factors rather than identifying predictors of this event. Clinical consensus on PE symptoms is lacking. Acute drops in lung function and changes in nutritional status have been recognized as key determinants [26]; however, relative contributions of these markers to prediction of PE onset have not been studied. Further, markers of growth/nutritional status (WFA, HFA and BMIp) and lung function (FEV_1_) are endogenous variables. In this epidemiologic study, we assessed predictive performance of these markers using a series of joint models. Our novel application confirmed the reliance of changes in the FEV_1_ trajectory on predicting PE events but revealed the clinical utility that these novel models could have for monitoring multiple indicators of disease progression for an individual patient. In parallel, our application highlights that, although pancreatic enzyme use is commonly employed as a marker of pancreatic insufficiency, their use was associated with lower PE frequency over follow-up (Table 1). It is possible that pancreatic enzyme use is also a marker of receiving higher-quality healthcare.

We found that the univariate joint model, which utilized FEV_1_ and PE event submodels without accounting for growth/nutritional status, produced the most accurate and precise predictions of all models considered. In addition, these predictions were largely robust across a range of age and prediction window strata. A potential reason for this finding is that the diagnosis of PE events may rely more heavily upon acute changes in lung function than changes in nutritional status. Changes in growth are relatively slow over time, compared to precipitous drops in lung function, which could be responsible for the lack of improved predictive performance when adding WFA, HFA or BMIp. Of the three markers available from the CFFPR to study growth/nutritional status, it is plausible that BMIp would be most likely to improve prediction of PE onset (after including FEV_1_); however, it appears that including BMIp as a submodel introduces additional noise into the joint modeling of these processes, thereby making predictions less accurate and more variable (Fig. 3). In addition, associations between clinical/demographic covariates and each outcome were largely consistent across FEV_1_ submodels. This finding further suggests that WFA, HFA and BMIp do not play a large role in clinical diagnosis of PE; however, the findings from the study aimed at prediction cannot serve as a substitute for assessing the causal effects of nutrition [27]. The results do warrant further investigation of a causal model examining the extent to which nutrition and growth mediate the relationship between lung function and PE onset. Understanding these causal underpinnings are a separate but important area of future work.

The multivariate joint models afforded the opportunity to obtain dynamic predictions at the individual patient level (Fig. 4); however, using multiple nutrition markers may decrease precision of PE predictions (Fig. 5). The decrease in precision may be due to relatively large variability in each outcome both between patients and within individual patients over time. Both Figs. 4 and 5 illustrate the substantial intra-individual variability in each of the lung function and growth markers. In contrast with joint modeling results, we found that patients who experienced a PE tended to have lower WFA, HFA and BMIp, as well as lower FEV_1_ (Table 1). If changes in nutrition markers are less influential in making a PE diagnosis than change in lung function, the contrasting results could reflect differences between association and prediction [27]. All of the outcome markers could be associated with PE onset but not all markers may be necessary to accurately predict it. The focus of the manuscript was to investigate the predictive performance of the different joint models. Hence, we did not assess the fit of those models. Using these dynamic predictions revealed the heterogeneous nature of CF disease progression but also highlighted the benefit of updated predictions across clinic visits. The ability to simultaneously monitor multiple markers of disease progression, which may serve as a clinical decision support tool, provides an advantage of the multivariate joint model over the univariate joint model and the more conventional Cox model. An area of future study could be the additional consideration of other markers of lung function as predictors, including forced vital capacity (FVC), the FEV_1_/FVC ratio and forced expiratory flow at 25–75% (referred to as FEF25–75). Furthermore, in this research we assumed the underlying value of the biomarkers (FEV_1,_ WFA, HFA and BMIp) to be associated with PE. A special feature of these biomarkers is that they are time-dependent, therefore the assumption of the underlying value might be too strict. Future research could focus on investigating whether other structures, such as the slope of these markers, could provide a stronger association with PE and a better predictive performance.

A limitation of this study is that the models cannot be used to draw conclusions about recurrent PE events, since the PE event submodel estimates time-to-first PE. Although this focus simplifies analyses, time-to-first PE during follow up tended to occur in early adulthood (Fig. 2). It is possible that a portion of patients experienced a PE prior to follow-up available in the data (e.g., patients born in older birth cohorts). In addition, the advent of newer therapies and/or quality improvement initiatives likely increase the overall trajectories of outcomes studied. We accounted for these potential sources of bias by including levels of birth cohort through linear and nonlinear terms in each of the longitudinal submodels (Tables S1-S5). Consequently, including higher-order associations through splines required additional degrees of freedom. Future work is needed on covariate selection strategies in the context of multivariate joint models [28, 29]. Fitting these more complex models to a large number of patients followed over long periods of time creates a computational challenge. We addressed this issue by partitioning into distinct sets of patients, which was the only feasible manner to estimate the model parameters; however, the bootstrap approach that we employed does not account for all the variability of the outcomes. Lastly, the multivariate joint models presented here allow for different patterns of missing data among the longitudinal outcomes but assume that the missingness is conditional on observed data only (i.e., missing at random), specifically occurrence of PE. It is possible that missingness could be related to patient severity. A recent study demonstrates that linear mixed effects modeling, which is the framework for the longitudinal submodels used in this application, can alleviate this potential source of bias [30].

## Conclusion

The univariate joint modeling of the CFFPR demonstrates the reliance of PE diagnosis on FEV_1_, and multivariate joint modeling of FEV_1_ and other outcomes related to nutritional status and growth can be used for routine medical monitoring of an individual patient and dynamic assessment of PE risk over time.

## Supplementary information

**Additional file 1: Table S1.** Joint Model with Longitudinal FEV1 and PE onset. **Table S2**. Joint Model with Longitudinal FEV1, BMIp and PE onset. **Table S3**. Joint Model with Longitudinal FEV1, HFA and PE onset. **Table S4**. Joint Model with Longitudinal FEV1, WFA and PE onset. **Table S5**. Joint Model with Longitudinal FEV1, WFA, HFA and PE onset.

**Additional file 2.** Multivariate joint modeling to identify markers of growth and lung function decline that predict cystic fibrosis pulmonary exacerbation onset.

## Data Availability

Implementation code for the models is provided as supplemental material (Supplementary File 2). In accordance with the CFFPR Information Use Agreement, the authors are not authorized to make the CF data accessible. Inquiries regarding data access may be directed via email to the Cystic Fibrosis Foundation: datarequests@cff.org.

## Abbreviations

AUC: Area under the receiver-operator characteristic curve
BMIp: Body mass index percentile
CF: Cystic fibrosis
CFF: CYSTIC Fibrosis Foundation
CFRD: Cystic fibrosis related diabetes
CI: Confidence interval
FEV_1_: Forced expiratory volume in 1 s of % predicted
HFA: Height-for-age percentile
MRSA: *Methicillin-resistant Staphylococcus aureus*
Pa: *Pseudomonas aeruginosa*
PE: Pulmonary exacerbation
SES: Socioeconomic status
WFA: Weight-for-age percentile
