# Supplementary material for: Multivariate joint modeling to identify markers of growth and lung function decline that predict cystic fibrosis pulmonary exacerbation onset
Source: BMC Pulm Med. 2020 May 19;20:142. doi: 10.1186/s12890-020-1177-z (PMC7236487; doi:10.1186/s12890-020-1177-z)
Supplement: Supplementary file 1 — Additional file 1: Table S1. Joint Model with Longitudinal FEV1 and PE onset. Table S2. Joint Model with Longitudinal FEV1, BMIp and PE onset. Table S3. Joint Model with Longitudinal FEV1, HFA and PE onset. Table S4. Joint Model with Longitudinal FEV1, WFA and PE onset. Table S5. Joint Model with Longitudinal FEV1, WFA, HFA and PE onset. [file 12890_2020_1177_MOESM1_ESM.docx]

**Supplemental Material**

**Multivariate joint modeling to identify markers of growth and lung function decline that predict cystic fibrosis pulmonary exacerbation onset**

**Andrinopoulou, E.R., Clancy, J.P., & Szczesniak, R.D.**

**Overview of Tables:** Results for each submodel are expressed as posterior means and standard errors (SEs) from each Gibbs sampler used to estimate the joint models.

**Abbreviations:**

**Table of Contents for Joint Models:**

Table S1: FEV1 and PE onset

Table S2: FEV1, BMIp and PE onset

Table S3: FEV1, HFA and PE onset

Table S4: FEV1, WFA and PE onset

Table S5: FEV1, WFA, HFA and PE onset

**Table S1: Joint Model with Longitudinal FEV1 and PE onset**

FEV1 Submodel:

|  | Parameter Estimate | SE | P-value |
| --- | --- | --- | --- |
| Intercept | 76.77 | 0.23 | <0.0001 |
| Age-related spline terms |  |  |  |
| $f_{1}(age)$ | -0.97 | 0.84 | 0.945 |
| $f_{2}(age)$ | 2.89 | 0.48 | 0.662 |
| $f_{3}(age)$ | -14.69 | 0.60 | 0.023 |
| Genotype |  |  |  |
| Heterozygous | 1.41 | 0.12 | 0.099 |
| None/Unknown | 1.13 | 0.60 | 0.519 |
| Ref: Homozygous |  |  |  |
| Male | 0.47 | 0.12 | 0.560 |
| Hispanic Ethnicity | -6.33 | 0.21 | <0.0001 |
| Low SES | -0.39 | 0.01 | 0.003 |
| Birth Cohort |  |  |  |
| 1981 – 1989 | 4.63 | 0.51 | 0.213 |
| 1990 – 1994 | 6.27 | 0.37 | 0.041 |
| 1995 – 1999 | 9.36 | 0.18 | <0.0001 |
| > 1999 | 11.52 | 0.21 | <0.0001 |
| Ref: < 1981 |  |  |  |
| MRSA | -0.90 | 0.01 | <0.0001 |
| Pa | -0.43 | 0.11 | 0.058 |
| CFRD | -1.24 | 0.20 | 0.005 |
| On pancreatic enzymes | 0.55 | 0.06 | <0.0001 |
| # of visits per year (rolling) | 0.04 | 0.01 | 0.231 |
| Heterozygous $\times f_{1}(age)$ | 0.25 | 0.76 | 0.904 |
| Heterozygous $\times f_{2}(age)$ | 1.41 | 0.12 | 0.396 |
| Heterozygous $\times f_{3}(age)$ | 1.88 | 0.46 | 0.238 |
| None/Unknown $\times f_{1}(age)$ | 2.10 | 0.67 | 0.330 |
| None/Unknown $\times f_{2}(age)$ | 4.66 | 1.88 | 0.321 |
| None/Unknown $\times f_{3}(age)$ | 6.01 | 0.19 | <0.0001 |
| Male $\times f_{1}(age)$ | 4.13 | 0.02 | <0.0001 |
| Male $\times f_{2}(age)$ | 1.52 | 0.70 | 0.525 |
| Male $\times f_{3}(age)$ | 2.12 | 0.55 | 0.187 |
| Hispanic Ethnicity $\times f_{1}(age)$ | -1.48 | 1.05 | 0.663 |
| Hispanic Ethnicity $\times f_{2}(age)$ | -0.23 | 0.10 | 0.932 |
| Hispanic Ethnicity $\times f_{3}(age)$ | 1.88 | 0.23 | 0.447 |
| Low SES $\times f_{1}(age)$ | -1.45 | 0.30 | 0.239 |
| Low SES$\times f_{2}(age)$ | -10.59 | 0.30 | <0.0001 |
| Low SES $\times f_{3}(age)$ | -4.25 | 0.04 | <0.0001 |
| (1981 – 1989) $\times f_{1}(age)$ | -2.18 | 0.23 | 0.524 |
| (1981 – 1989) $\times f_{2}(age)$ | -8.15 | 2.13 | 0.372 |
| (1981 – 1989) $\times f_{3}(age)$ | -0.02 | 0.35 | 0.981 |
| (1990 – 1994) $\times f_{1}(age)$ | -1.11 | 0.57 | 0.792 |
| (1990 – 1994) $\times f_{2}(age)$ | -7.53 | 0.27 | 0.114 |
| (1990 – 1994) $\times f_{3}(age)$ | 1.37 | 0.68 | 0.805 |
| (1995 – 1999) $\times f_{1}(age)$ | -0.16 | 0.53 | 0.975 |
| (1995 – 1999) $\times f_{2}(age)$ | -6.50 | 0.48 | 0.232 |
| (1995 – 1999) $\times f_{3}(age)$ | 3.47 | 0.17 | 0.329 |
| (> 1999) $\times f_{1}(age)$ | -2.76 | 1.39 | 0.736 |
| (> 1999) $\times f_{2}(age)$ | -5.67 | 0.33 | 0.408 |
| (> 1999) $\times f_{3}(age)$ | 3.33 | 0.45 | 0.700 |
| MRSA$\times f_{1}(age)$ | -1.86 | 0.57 | 0.463 |
| MRSA $\times f_{2}(age)$ | -7.56 | 1.05 | 0.006 |
| MRSA $\times f_{3}(age)$ | -3.29 | 1.49 | 0.457 |
| Pa$\times f_{1}(age)$ | -2.07 | 0.34 | 0.145 |
| Pa $\times f_{2}(age)$ | -9.77 | 0.87 | <0.0001 |
| Pa $\times f_{3}(age)$ | -5.66 | 0.65 | <0.0001 |
| CFRD$\times f_{1}(age)$ | -3.11 | 0.21 | 0.302 |
| CFRD $\times f_{2}(age)$ | -13.39 | 1.75 | <0.0001 |
| CFRD $\times f_{3}(age)$ | -5.17 | 1.48 | 0.153 |
| $On pancreatic enzymes$ $\times f_{1}(age)$ | -0.52 | 0.02 | 0.663 |
| On pancreatic enzymes $\times f_{2}(age)$ | 0.00 | 0.07 | 0.983 |
| On pancreatic enzymes $\times f_{3}(age)$ | 0.97 | 0.23 | 0.576 |
| $\# of visits per year (rolling)$ $\times f_{1}(age)$ | 0.36 | 0.49 | 0.983 |
| # of visits per year (rolling)$\times f_{2}(age)$ | 0.52 | 0.42 | 0.935 |
| # of visits per year (rolling)$\times f_{3}(age)$ | -6.16 | 0.42 | 0.257 |

Survival Submodel:

|  | Parameter Estimate | SE | P-value |
| --- | --- | --- | --- |
| Low SES | 0.33 | 0.01 | <0.0001 |
| Male | -0.21 | 0.01 | 0.001 |
| On pancreatic enzymes | -0.18 | 0.00 | 0.001 |
| Pa | -0.31 | 0.01 | <0.0001 |
| MRSA | 0.27 | 0.00 | <0.0001 |
| CFRD | -0.28 | 0.01 | 0.011 |

Survival (association parameters):

|  | Alpha coefficient | SE | P-value |
| --- | --- | --- | --- |
| FEV1 value | -0.0305 | 6e-04 | 0.001 |

**Table S2: Joint Model with Longitudinal FEV1, BMIp and PE onset**

FEV1 Submodel:

|  | Parameter Estimate | SE | P-value |
| --- | --- | --- | --- |
| Intercept | 77.26 | 0.36 | <0.0001 |
| Age-related spline terms |  |  |  |
| $f_{1}(age)$ | -1.59 | 0.93 | 0.883 |
| $f_{2}(age)$ | 4.12 | 0.53 | 0.532 |
| $f_{3}(age)$ | -13.40 | 0.90 | 0.064 |
| Genotype |  |  |  |
| Heterozygous | 1.44 | 0.10 | 0.077 |
| None/Unknown | 1.20 | 0.55 | 0.475 |
| Ref: Homozygous |  |  |  |
| Male | 0.43 | 0.10 | 0.571 |
| Hispanic Ethnicity | 6.46 | 0.17 | <0.0001 |
| Low SES | -0.39 | 0.01 | 0.011 |
| Birth Cohort |  |  |  |
| 1981 – 1989 | 3.99 | 0.65 | 0.307 |
| 1990 – 1994 | 5.68 | 0.28 | 0.037 |
| 1995 – 1999 | 8.70 | 0.24 | 0.002 |
| > 1999 | 10.84 | 0.18 | 0.001 |
| Ref: < 1981 |  |  |  |
| MRSA | -0.93 | 0.01 | <0.0001 |
| Pa | -0.39 | 0.08 | 0.096 |
| CFRD | -1.16 | 0.21 | 0.011 |
| On pancreatic enzymes | 0.58 | 0.07 | <0.0001 |
| # of visits per year (rolling) | 0.06 | 0.01 | 0.095 |
| Heterozygous $\times f_{1}(age)$ | 0.21 | 0.70 | 0.935 |
| Heterozygous $\times f_{2}(age)$ | 1.39 | 0.12 | 0.407 |
| Heterozygous $\times f_{3}(age)$ | 1.76 | 0.48 | 0.284 |
| None/Unknown $\times f_{1}(age)$ | 2.03 | 0.50 | 0.313 |
| None/Unknown $\times f_{2}(age)$ | 4.47 | 1.64 | 0.331 |
| None/Unknown $\times f_{3}(age)$ | 5.60 | 0.22 | 0.001 |
| Male $\times f_{1}(age)$ | 4.66 | 0.04 | <0.0001 |
| Male $\times f_{2}(age)$ | 1.51 | 0.66 | 0.466 |
| Male $\times f_{3}(age)$ | 1.87 | 0.42 | 0.209 |
| Hispanic Ethnicity $\times f_{1}(age)$ | -1.48 | 0.87 | 0.649 |
| Hispanic Ethnicity $\times f_{2}(age)$ | -0.33 | 0.19 | 0.909 |
| Hispanic Ethnicity $\times f_{3}(age)$ | 1.82 | 0.24 | 0.495 |
| Low SES $\times f_{1}(age)$ | -1.46 | 0.26 | 0.219 |
| Low SES$\times f_{2}(age)$ | -10.83 | 0.36 | <0.0001 |
| Low SES $\times f_{3}(age)$ | -4.66 | 0.06 | <0.0001 |
| (1981 – 1989) $\times f_{1}(age)$ | -2.05 | 0.26 | 0.482 |
| (1981 – 1989) $\times f_{2}(age)$ | -7.17 | 1.83 | 0.405 |
| (1981 – 1989) $\times f_{3}(age)$ | 0.60 | 0.37 | 0.849 |
| (1990 – 1994) $\times f_{1}(age)$ | -1.20 | 0.36 | 0.722 |
| (1990 – 1994) $\times f_{2}(age)$ | -6.86 | 0.38 | 0.171 |
| (1990 – 1994) $\times f_{3}(age)$ | 2.00 | 0.94 | 0.651 |
| (1995 – 1999) $\times f_{1}(age)$ | -0.09 | 0.45 | 0.979 |
| (1995 – 1999) $\times f_{2}(age)$ | -5.90 | 0.26 | 0.255 |
| (1995 – 1999) $\times f_{3}(age)$ | 3.93 | 0.26 | 0.283 |
| (> 1999) $\times f_{1}(age)$ | -2.23 | 1.11 | 0.767 |
| (> 1999) $\times f_{2}(age)$ | -5.35 | 0.36 | 0.417 |
| (> 1999) $\times f_{3}(age)$ | 3.15 | 0.51 | 0.705 |
| MRSA$\times f_{1}(age)$ | -1.80 | 0.50 | 0.472 |
| MRSA $\times f_{2}(age)$ | -7.55 | 0.93 | 0.006 |
| MRSA $\times f_{3}(age)$ | -3.43 | 1.51 | 0.415 |
| Pa$\times f_{1}(age)$ | -1.64 | 0.34 | 0.275 |
| Pa $\times f_{2}(age)$ | -9.94 | 0.85 | <0.0001 |
| Pa $\times f_{3}(age)$ | -5.86 | 0.76 | 0.001 |
| CFRD$\times f_{1}(age)$ | -2.71 | 0.32 | 0.368 |
| CFRD $\times f_{2}(age)$ | -13.07 | 1.79 | 0.001 |
| CFRD $\times f_{3}(age)$ | -5.20 | 1.46 | 0.161 |
| $On pancreatic enzymes$ $\times f_{1}(age)$ | -0.71 | 0.02 | 0.572 |
| On pancreatic enzymes $\times f_{2}(age)$ | 0.36 | 0.16 | 0.853 |
| On pancreatic enzymes $\times f_{3}(age)$ | 1.47 | 0.30 | 0.432 |
| $\# of visits per year (rolling)$ $\times f_{1}(age)$ | 0.33 | 0.64 | 0.973 |
| # of visits per year (rolling)$\times f_{2}(age)$ | -1.67 | 0.48 | 0.811 |
| # of visits per year (rolling)$\times f_{3}(age)$ | -7.77 | 0.67 | 0.185 |

BMI Submodel:

|  | Parameter Estimate | SE | P-value |
| --- | --- | --- | --- |
| Intercept | 47.75 | 0.74 | <0.0001 |
| Age-related spline terms |  |  |  |
| $f_{1}(age)$ | 3.64 | 0.72 | 0.679 |
| $f_{2}(age)$ | -10.96 | 0.43 | 0.155 |
| $f_{3}(age)$ | -3.72 | 0.44 | 0.583 |
| Genotype |  |  |  |
| Heterozygous | 1.01 | 0.47 | 0.488 |
| None/Unknown | 0.84 | 0.18 | 0.635 |
| Ref: Homozygous |  |  |  |
| Male | 2.04 | 0.39 | 0.127 |
| Hispanic Ethnicity | 0.53 | 0.48 | 0.836 |
| Low SES | 0.05 | 0.25 | 0.689 |
| Birth Cohort |  |  |  |
| 1981 – 1989 | 18.65 | 1.09 | <0.0001 |
| 1990 – 1994 | 4.02 | 0.22 | 0.180 |
| 1995 – 1999 | 4.83 | 0.30 | 0.113 |
| > 1999 | 7.05 | 0.14 | 0.009 |
| Ref: < 1981 |  |  |  |
| MRSA | -0.32 | 0.25 | 0.653 |
| Pa | 0.27 | 0.02 | 0.151 |
| CFRD | 0.14 | 0.03 | 0.678 |
| On pancreatic enzymes | 0.32 | 0.03 | 0.013 |
| # of visits per year (rolling) | 0.29 | 0.03 | <0.0001 |
| Heterozygous $\times f_{1}(age)$ | 2.44 | 1.06 | 0.357 |
| Heterozygous $\times f_{2}(age)$ | 5.23 | 0.36 | 0.033 |
| Heterozygous $\times f_{3}(age)$ | 1.36 | 0.80 | 0.639 |
| None/Unknown $\times f_{1}(age)$ | 4.59 | 0.04 | 0.050 |
| None/Unknown $\times f_{2}(age)$ | 10.00 | 0.30 | 0.005 |
| None/Unknown $\times f_{3}(age)$ | 4.88 | 0.70 | 0.093 |
| Male $\times f_{1}(age)$ | -11.27 | 0.52 | <0.0001 |
| Male $\times f_{2}(age)$ | -5.24 | 0.86 | 0.066 |
| Male $\times f_{3}(age)$ | -11.70 | 0.38 | <0.0001 |
| Hispanic Ethnicity $\times f_{1}(age)$ | 2.11 | 1.16 | 0.630 |
| Hispanic Ethnicity $\times f_{2}(age)$ | -5.10 | 0.12 | 0.155 |
| Hispanic Ethnicity $\times f_{3}(age)$ | 1.13 | 0.11 | 0.711 |
| Low SES $\times f_{1}(age)$ | -0.54 | 0.15 | 0.746 |
| Low SES$\times f_{2}(age)$ | -5.05 | 0.10 | 0.004 |
| Low SES $\times f_{3}(age)$ | -4.12 | 0.30 | 0.021 |
| (1981 – 1989) $\times f_{1}(age)$ | -14.35 | 0.12 | <0.0001 |
| (1981 – 1989) $\times f_{2}(age)$ | -36.84 | 3.11 | <0.0001 |
| (1981 – 1989) $\times f_{3}(age)$ | -3.29 | 0.10 | 0.287 |
| (1990 – 1994) $\times f_{1}(age)$ | -8.97 | 0.68 | 0.029 |
| (1990 – 1994) $\times f_{2}(age)$ | -0.50 | 0.18 | 0.899 |
| (1990 – 1994) $\times f_{3}(age)$ | 5.75 | 0.71 | 0.176 |
| (1995 – 1999) $\times f_{1}(age)$ | -8.98 | 0.13 | 0.009 |
| (1995 – 1999) $\times f_{2}(age)$ | 16.90 | 1.31 | 0.005 |
| (1995 – 1999) $\times f_{3}(age)$ | 20.32 | 1.65 | 0.001 |
| (> 1999) $\times f_{1}(age)$ | 3.94 | 0.35 | 0.633 |
| (> 1999) $\times f_{2}(age)$ | 8.50 | 0.22 | 0.203 |
| (> 1999) $\times f_{3}(age)$ | -4.86 | 0.35 | 0.587 |
| MRSA$\times f_{1}(age)$ | -2.99 | 0.28 | 0.349 |
| MRSA $\times f_{2}(age)$ | -1.45 | 1.00 | 0.742 |
| MRSA $\times f_{3}(age)$ | -2.22 | 0.19 | 0.529 |
| Pa$\times f_{1}(age)$ | -0.66 | 0.88 | 0.751 |
| Pa $\times f_{2}(age)$ | -9.72 | 0.59 | <0.0001 |
| Pa $\times f_{3}(age)$ | -4.60 | 0.95 | 0.064 |
| CFRD$\times f_{1}(age)$ | 0.38 | 0.18 | 0.943 |
| CFRD $\times f_{2}(age)$ | -7.96 | 2.61 | 0.191 |
| CFRD $\times f_{3}(age)$ | -2.48 | 0.60 | 0.555 |
| $On pancreatic enzymes$ $\times f_{1}(age)$ | -3.33 | 0.48 | 0.132 |
| On pancreatic enzymes $\times f_{2}(age)$ | -5.61 | 1.05 | 0.041 |
| On pancreatic enzymes $\times f_{3}(age)$ | 1.88 | 0.25 | 0.400 |
| $\# of visits per year (rolling)$ $\times f_{1}(age)$ | 1.47 | 0.39 | 0.812 |
| # of visits per year (rolling)$\times f_{2}(age)$ | -0.98 | 0.63 | 0.910 |
| # of visits per year (rolling)$\times f_{3}(age)$ | -2.54 | 0.44 | 0.715 |

Survival Submodel:

|  | Parameter Estimate | SE | P-value |
| --- | --- | --- | --- |
| Low SES | 0.32 | 0.01 | <0.0001 |
| Male | -0.20 | 0.01 | 0.002 |
| On pancreatic enzymes | -0.18 | 0.00 | 0.007 |
| Pa | -0.32 | 0.01 | <0.0001 |
| MRSA | 0.26 | 0.01 | <0.0001 |
| CFRD | -0.29 | 0.01 | 0.004 |

Survival (association parameters):

|  | Alpha coefficient | SE | P-value |
| --- | --- | --- | --- |
| FEV1 value | -0.0324 | 6e-04 | 0.002 |
| BMIp value | -0.0004 | 3e-04 | 0.988 |

**Table S3: Joint Model with Longitudinal FEV1, HFA and PE onset**

FEV1 Submodel:

|  | Parameter Estimate | SE | P-value |
| --- | --- | --- | --- |
| Intercept | 77.03 | 0.32 | <0.0001 |
| Age-related spline terms |  |  |  |
| $f_{1}(age)$ | -1.82 | 0.77 | 0.844 |
| $f_{2}(age)$ | 2.42 | 0.59 | 0.727 |
| $f_{3}(age)$ | -14.10 | 0.68 | 0.037 |
| Genotype |  |  |  |
| Heterozygous | 1.41 | 0.09 | 0.081 |
| None/Unknown | 1.12 | 0.62 | 0.525 |
| Ref: Homozygous |  |  |  |
| Male | 0.49 | 0.13 | 0.539 |
| Hispanic Ethnicity | 6.28 | 0.30 | <0.0001 |
| Low SES | -0.36 | 0.01 | 0.017 |
| Birth Cohort |  |  |  |
| 1981 – 1989 | 4.34 | 0.38 | 0.229 |
| 1990 – 1994 | 6.02 | 0.22 | 0.035 |
| 1995 – 1999 | 9.09 | 0.19 | <0.0001 |
| > 1999 | 11.23 | 0.20 | <0.0001 |
| Ref: < 1981 |  |  |  |
| MRSA | -0.91 | 0.01 | <0.0001 |
| Pa | -0.44 | 0.09 | 0.049 |
| CFRD | -1.21 | 0.26 | 0.007 |
| On pancreatic enzymes | 0.56 | 0.08 | <0.0001 |
| # of visits per year (rolling) | 0.04 | 0.01 | 0.257 |
| Heterozygous $\times f_{1}(age)$ | 0.30 | 0.73 | 0.870 |
| Heterozygous $\times f_{2}(age)$ | 1.55 | 0.12 | 0.366 |
| Heterozygous $\times f_{3}(age)$ | 2.07 | 0.49 | 0.203 |
| None/Unknown $\times f_{1}(age)$ | 2.06 | 0.72 | 0.313 |
| None/Unknown $\times f_{2}(age)$ | 4.80 | 1.78 | 0.315 |
| None/Unknown $\times f_{3}(age)$ | 6.22 | 0.25 | <0.0001 |
| Male $\times f_{1}(age)$ | 3.84 | 0.02 | <0.0001 |
| Male $\times f_{2}(age)$ | 1.62 | 0.87 | 0.505 |
| Male $\times f_{3}(age)$ | 2.16 | 0.57 | 0.147 |
| Hispanic Ethnicity $\times f_{1}(age)$ | -1.27 | 1.04 | 0.673 |
| Hispanic Ethnicity $\times f_{2}(age)$ | -0.33 | 0.14 | 0.916 |
| Hispanic Ethnicity $\times f_{3}(age)$ | 1.59 | 0.24 | 0.531 |
| Low SES $\times f_{1}(age)$ | -1.54 | 0.29 | 0.182 |
| Low SES$\times f_{2}(age)$ | -10.22 | 0.17 | <0.0001 |
| Low SES $\times f_{3}(age)$ | -4.05 | 0.18 | <0.0001 |
| (1981 – 1989) $\times f_{1}(age)$ | -1.98 | 0.22 | 0.541 |
| (1981 – 1989) $\times f_{2}(age)$ | -8.03 | 1.89 | 0.354 |
| (1981 – 1989) $\times f_{3}(age)$ | 0.17 | 0.22 | 0.963 |
| (1990 – 1994) $\times f_{1}(age)$ | -1.13 | 0.36 | 0.767 |
| (1990 – 1994) $\times f_{2}(age)$ | -7.36 | 0.28 | 0.144 |
| (1990 – 1994) $\times f_{3}(age)$ | 1.62 | 0.67 | 0.743 |
| (1995 – 1999) $\times f_{1}(age)$ | -0.04 | 0.33 | 0.962 |
| (1995 – 1999) $\times f_{2}(age)$ | -6.03 | 0.27 | 0.221 |
| (1995 – 1999) $\times f_{3}(age)$ | 4.29 | 0.20 | 0.244 |
| (> 1999) $\times f_{1}(age)$ | -2.63 | 1.24 | 0.781 |
| (> 1999) $\times f_{2}(age)$ | -5.68 | 0.31 | 0.397 |
| (> 1999) $\times f_{3}(age)$ | 3.37 | 0.50 | 0.700 |
| MRSA$\times f_{1}(age)$ | -1.78 | 0.76 | 0.485 |
| MRSA $\times f_{2}(age)$ | -7.76 | 1.20 | 0.005 |
| MRSA $\times f_{3}(age)$ | -3.43 | 1.64 | 0.415 |
| Pa$\times f_{1}(age)$ | -2.12 | 0.32 | 0.131 |
| Pa $\times f_{2}(age)$ | -9.71 | 0.92 | <0.0001 |
| Pa $\times f_{3}(age)$ | -5.59 | 0.64 | <0.0001 |
| CFRD$\times f_{1}(age)$ | -3.40 | 0.17 | 0.260 |
| CFRD $\times f_{2}(age)$ | -13.04 | 1.65 | 0.001 |
| CFRD $\times f_{3}(age)$ | -4.99 | 1.43 | 0.188 |
| $On pancreatic enzymes$ $\times f_{1}(age)$ | -0.38 | 0.02 | 0.745 |
| On pancreatic enzymes $\times f_{2}(age)$ | 0.15 | 0.07 | 0.931 |
| On pancreatic enzymes $\times f_{3}(age)$ | 0.85 | 0.16 | 0.586 |
| $\# of visits per year (rolling)$ $\times f_{1}(age)$ | 1.14 | 0.58 | 0.866 |
| # of visits per year (rolling)$\times f_{2}(age)$ | 0.49 | 0.46 | 0.921 |
| # of visits per year (rolling)$\times f_{3}(age)$ | -7.04 | 0.49 | 0.157 |

HFA Submodel:

|  | Parameter Estimate | SE | P-value |
| --- | --- | --- | --- |
| Intercept | 15.55 | 0.11 | <0.0001 |
| Age-related spline terms |  |  |  |
| $f_{1}(age)$ | 10.01 | 0.34 | 0.128 |
| $f_{2}(age)$ | -0.75 | 0.35 | 0.913 |
| $f_{3}(age)$ | 10.75 | 1.05 | 0.185 |
| Genotype |  |  |  |
| Heterozygous | 3.62 | 0.12 | <0.0001 |
| None/Unknown | 1.78 | 0.50 | 0.385 |
| Ref: Homozygous |  |  |  |
| Male | 2.43 | 0.78 | 0.223 |
| Hispanic Ethnicity | 6.57 | 0.10 | <0.0001 |
| Low SES | -0.06 | 0.05 | 0.630 |
| Birth Cohort |  |  |  |
| 1981 – 1989 | -1.03 | 0.12 | 0.697 |
| 1990 – 1994 | 5.70 | 0.29 | 0.047 |
| 1995 – 1999 | 11.90 | 0.43 | <0.0001 |
| > 1999 | 18.40 | 0.89 | <0.0001 |
| Ref: < 1981 |  |  |  |
| MRSA | -0.19 | 0.10 | 0.368 |
| Pa | 0.04 | 0.11 | 0.745 |
| CFRD | 0.10 | 0.20 | 0.887 |
| On pancreatic enzymes | 0.01 | 0.02 | 0.888 |
| # of visits per year (rolling) | -0.11 | 0.01 | <0.0001 |
| Heterozygous $\times f_{1}(age)$ | -0.46 | 0.38 | 0.812 |
| Heterozygous $\times f_{2}(age)$ | 1.03 | 0.08 | 0.649 |
| Heterozygous $\times f_{3}(age)$ | 0.12 | 0.45 | 0.959 |
| None/Unknown $\times f_{1}(age)$ | 2.02 | 0.89 | 0.490 |
| None/Unknown $\times f_{2}(age)$ | 2.77 | 0.20 | 0.381 |
| None/Unknown $\times f_{3}(age)$ | -3.97 | 0.29 | 0.211 |
| Male $\times f_{1}(age)$ | -1.32 | 1.04 | 0.585 |
| Male $\times f_{2}(age)$ | -7.89 | 1.77 | 0.021 |
| Male $\times f_{3}(age)$ | -6.31 | 0.42 | 0.012 |
| Hispanic Ethnicity $\times f_{1}(age)$ | 1.63 | 2.08 | 0.893 |
| Hispanic Ethnicity $\times f_{2}(age)$ | 4.19 | 0.24 | 0.229 |
| Hispanic Ethnicity $\times f_{3}(age)$ | 11.01 | 0.36 | 0.003 |
| Low SES $\times f_{1}(age)$ | -2.20 | 1.06 | 0.391 |
| Low SES$\times f_{2}(age)$ | -8.72 | 1.05 | <0.0001 |
| Low SES $\times f_{3}(age)$ | -8.22 | 1.08 | 0.001 |
| (1981 – 1989) $\times f_{1}(age)$ | -7.65 | 1.85 | 0.165 |
| (1981 – 1989) $\times f_{2}(age)$ | 17.81 | 0.94 | 0.002 |
| (1981 – 1989) $\times f_{3}(age)$ | -4.15 | 1.10 | 0.337 |
| (1990 – 1994) $\times f_{1}(age)$ | -7.94 | 2.32 | 0.207 |
| (1990 – 1994) $\times f_{2}(age)$ | 6.74 | 0.61 | 0.221 |
| (1990 – 1994) $\times f_{3}(age)$ | -13.36 | 3.12 | 0.011 |
| (1995 – 1999) $\times f_{1}(age)$ | -1.23 | 2.52 | 0.910 |
| (1995 – 1999) $\times f_{2}(age)$ | -9.54 | 0.59 | 0.086 |
| (1995 – 1999) $\times f_{3}(age)$ | -40.32 | 0.88 | <0.0001 |
| (> 1999) $\times f_{1}(age)$ | 28.02 | 0.25 | <0.0001 |
| (> 1999) $\times f_{2}(age)$ | 12.68 | 0.63 | 0.060 |
| (> 1999) $\times f_{3}(age)$ | -8.45 | 0.24 | 0.345 |
| MRSA$\times f_{1}(age)$ | -1.08 | 0.68 | 0.766 |
| MRSA $\times f_{2}(age)$ | -2.01 | 0.49 | 0.602 |
| MRSA $\times f_{3}(age)$ | 0.79 | 1.04 | 0.837 |
| Pa$\times f_{1}(age)$ | -2.27 | 0.45 | 0.273 |
| Pa $\times f_{2}(age)$ | -1.79 | 0.91 | 0.541 |
| Pa $\times f_{3}(age)$ | 0.68 | 0.11 | 0.771 |
| CFRD$\times f_{1}(age)$ | -11.74 | 1.32 | 0.008 |
| CFRD $\times f_{2}(age)$ | -9.78 | 0.08 | 0.012 |
| CFRD $\times f_{3}(age)$ | -4.43 | 0.24 | 0.329 |
| $On pancreatic enzymes$ $\times f_{1}(age)$ | 1.50 | 0.42 | 0.457 |
| On pancreatic enzymes $\times f_{2}(age)$ | -3.91 | 1.92 | 0.355 |
| On pancreatic enzymes $\times f_{3}(age)$ | -1.08 | 0.60 | 0.711 |
| $\# of visits per year (rolling)$ $\times f_{1}(age)$ | 4.82 | 0.39 | 0.448 |
| # of visits per year (rolling)$\times f_{2}(age)$ | 8.10 | 0.67 | 0.259 |
| # of visits per year (rolling)$\times f_{3}(age)$ | 3.43 | 0.64 | 0.610 |

Survival Submodel:

|  | Parameter Estimate | SE | P-value |
| --- | --- | --- | --- |
| Low SES | 0.33 | 0.01 | <0.0001 |
| Male | -0.20 | 0.01 | 0.002 |
| On pancreatic enzymes | -0.18 | 0.00 | 0.005 |
| Pa | -0.30 | 0.01 | <0.0001 |
| MRSA | 0.26 | 0.00 | <0.0001 |
| CFRD | -0.27 | 0.01 | 0.001 |

Survival (association parameters):

|  | Alpha coefficient | SE | P-value |
| --- | --- | --- | --- |
| FEV1 value | -0.0308 | 5e-04 | <0.0001 |
| HFA value | 0.0004 | 1e-04 | 0.927 |

**Table S4: Joint Model with Longitudinal FEV1, WFA and PE onset**

FEV1 Submodel:

|  | Parameter Estimate | SE | P-value |
| --- | --- | --- | --- |
| Intercept | 77.29 | 0.24 | <0.0001 |
| Age-related spline terms |  |  |  |
| $f_{1}(age)$ | -2.14 | 0.89 | 0.806 |
| $f_{2}(age)$ | 3.78 | 0.55 | 0.576 |
| $f_{3}(age)$ | -14.16 | 0.84 | 0.052 |
| Genotype |  |  |  |
| Heterozygous | 1.44 | 0.13 | 0.080 |
| None/Unknown | 1.12 | 0.63 | 0.533 |
| Ref: Homozygous |  |  |  |
| Male | 0.48 | 0.09 | 0.529 |
| Hispanic Ethnicity | 6.35 | 0.20 | <0.0001 |
| Low SES | -0.35 | 0.01 | 0.018 |
| Birth Cohort |  |  |  |
| 1981 – 1989 | 4.21 | 0.67 | 0.279 |
| 1990 – 1994 | 5.72 | 0.21 | 0.035 |
| 1995 – 1999 | 8.73 | 0.18 | 0.001 |
| > 1999 | 10.86 | 0.18 | <0.0001 |
| Ref: < 1981 |  |  |  |
| MRSA | -0.92 | 0.01 | <0.0001 |
| Pa | -0.39 | 0.09 | 0.077 |
| CFRD | -1.08 | 0.31 | 0.040 |
| On pancreatic enzymes | 0.59 | 0.07 | <0.0001 |
| # of visits per year (rolling) | 0.05 | 0.01 | 0.115 |
| Heterozygous $\times f_{1}(age)$ | 0.26 | 0.80 | 0.903 |
| Heterozygous $\times f_{2}(age)$ | 1.53 | 0.17 | 0.390 |
| Heterozygous $\times f_{3}(age)$ | 1.84 | 0.53 | 0.263 |
| None/Unknown $\times f_{1}(age)$ | 2.15 | 0.48 | 0.284 |
| None/Unknown $\times f_{2}(age)$ | 4.72 | 2.40 | 0.347 |
| None/Unknown $\times f_{3}(age)$ | 5.71 | 0.25 | 0.002 |
| Male $\times f_{1}(age)$ | 4.37 | 0.03 | <0.0001 |
| Male $\times f_{2}(age)$ | 1.58 | 0.47 | 0.464 |
| Male $\times f_{3}(age)$ | 2.33 | 0.30 | 0.097 |
| Hispanic Ethnicity $\times f_{1}(age)$ | -1.45 | 0.96 | 0.642 |
| Hispanic Ethnicity $\times f_{2}(age)$ | -0.09 | 0.16 | 0.993 |
| Hispanic Ethnicity $\times f_{3}(age)$ | 1.89 | 0.24 | 0.501 |
| Low SES $\times f_{1}(age)$ | -1.38 | 0.34 | 0.275 |
| Low SES$\times f_{2}(age)$ | -10.60 | 0.33 | <0.0001 |
| Low SES $\times f_{3}(age)$ | -4.66 | 0.05 | <0.0001 |
| (1981 – 1989) $\times f_{1}(age)$ | -2.08 | 0.19 | 0.489 |
| (1981 – 1989) $\times f_{2}(age)$ | -7.76 | 1.98 | 0.434 |
| (1981 – 1989) $\times f_{3}(age)$ | 0.28 | 0.20 | 0.942 |
| (1990 – 1994) $\times f_{1}(age)$ | -1.42 | 0.45 | 0.683 |
| (1990 – 1994) $\times f_{2}(age)$ | -6.83 | 0.36 | 0.157 |
| (1990 – 1994) $\times f_{3}(age)$ | 1.96 | 0.76 | 0.683 |
| (1995 – 1999) $\times f_{1}(age)$ | -0.39 | 0.25 | 0.897 |
| (1995 – 1999) $\times f_{2}(age)$ | -5.57 | 0.36 | 0.254 |
| (1995 – 1999) $\times f_{3}(age)$ | 4.58 | 0.22 | 0.221 |
| (> 1999) $\times f_{1}(age)$ | -3.42 | 0.95 | 0.703 |
| (> 1999) $\times f_{2}(age)$ | -5.29 | 0.31 | 0.423 |
| (> 1999) $\times f_{3}(age)$ | 3.92 | 0.51 | 0.655 |
| MRSA$\times f_{1}(age)$ | -1.97 | 0.62 | 0.435 |
| MRSA $\times f_{2}(age)$ | -7.42 | 0.90 | 0.007 |
| MRSA $\times f_{3}(age)$ | -3.12 | 1.54 | 0.461 |
| Pa$\times f_{1}(age)$ | -1.71 | 0.43 | 0.261 |
| Pa $\times f_{2}(age)$ | -9.90 | 0.85 | <0.0001 |
| Pa $\times f_{3}(age)$ | -5.81 | 0.74 | 0.001 |
| CFRD$\times f_{1}(age)$ | -2.74 | 0.16 | 0.352 |
| CFRD $\times f_{2}(age)$ | -12.79 | 1.73 | <0.0001 |
| CFRD $\times f_{3}(age)$ | -5.06 | 1.34 | 0.195 |
| $On pancreatic enzymes$ $\times f_{1}(age)$ | -0.85 | 0.03 | 0.513 |
| On pancreatic enzymes $\times f_{2}(age)$ | 0.47 | 0.17 | 0.755 |
| On pancreatic enzymes $\times f_{3}(age)$ | 1.42 | 0.49 | 0.497 |
| $\# of visits per year (rolling)$ $\times f_{1}(age)$ | 1.03 | 0.67 | 0.908 |
| # of visits per year (rolling)$\times f_{2}(age)$ | -1.77 | 0.45 | 0.787 |
| # of visits per year (rolling)$\times f_{3}(age)$ | -7.32 | 0.62 | 0.199 |

WFA Submodel:

|  | Parameter Estimate | SE | P-value |
| --- | --- | --- | --- |
| Intercept | 34.09 | 0.14 | <0.0001 |
| Age-related spline terms |  |  |  |
| $f_{1}(age)$ | 3.99 | 0.74 | 0.608 |
| $f_{2}(age)$ | -9.01 | 0.41 | 0.224 |
| $f_{3}(age)$ | -1.33 | 0.40 | 0.849 |
| Genotype |  |  |  |
| Heterozygous | 3.20 | 0.19 | 0.007 |
| None/Unknown | 2.46 | 0.42 | 0.202 |
| Ref: Homozygous |  |  |  |
| Male | 0.60 | 0.49 | 0.675 |
| Hispanic Ethnicity | 4.21 | 0.26 | 0.031 |
| Low SES | 0.00 | 0.16 | 0.749 |
| Birth Cohort |  |  |  |
| 1981 – 1989 | 21.07 | 0.85 | <0.0001 |
| 1990 – 1994 | 1.74 | 0.20 | 0.563 |
| 1995 – 1999 | 3.38 | 0.46 | 0.297 |
| > 1999 | 7.37 | 0.49 | 0.023 |
| Ref: < 1981 |  |  |  |
| MRSA | -0.24 | 0.29 | 0.775 |
| Pa | 0.34 | 0.05 | 0.055 |
| CFRD | 0.11 | 0.08 | 0.731 |
| On pancreatic enzymes | 0.19 | 0.01 | 0.032 |
| # of visits per year (rolling) | 0.24 | 0.02 | <0.0001 |
| Heterozygous $\times f_{1}(age)$ | 1.51 | 0.35 | 0.431 |
| Heterozygous $\times f_{2}(age)$ | 3.69 | 0.31 | 0.127 |
| Heterozygous $\times f_{3}(age)$ | 1.28 | 0.61 | 0.616 |
| None/Unknown $\times f_{1}(age)$ | 4.55 | 0.22 | 0.054 |
| None/Unknown $\times f_{2}(age)$ | 8.13 | 0.59 | 0.021 |
| None/Unknown $\times f_{3}(age)$ | 2.67 | 0.65 | 0.408 |
| Male $\times f_{1}(age)$ | -9.75 | 0.31 | <0.0001 |
| Male $\times f_{2}(age)$ | 0.20 | 1.44 | 0.918 |
| Male $\times f_{3}(age)$ | -6.23 | 0.54 | 0.004 |
| Hispanic Ethnicity $\times f_{1}(age)$ | 2.92 | 2.10 | 0.625 |
| Hispanic Ethnicity $\times f_{2}(age)$ | -2.26 | 0.40 | 0.569 |
| Hispanic Ethnicity $\times f_{3}(age)$ | 6.35 | 0.48 | 0.093 |
| Low SES $\times f_{1}(age)$ | -1.64 | 0.05 | 0.259 |
| Low SES$\times f_{2}(age)$ | -7.35 | 0.30 | <0.0001 |
| Low SES $\times f_{3}(age)$ | -6.63 | 0.33 | 0.001 |
| (1981 – 1989) $\times f_{1}(age)$ | -13.26 | 0.10 | <0.0001 |
| (1981 – 1989) $\times f_{2}(age)$ | -44.45 | 2.68 | <0.0001 |
| (1981 – 1989) $\times f_{3}(age)$ | -4.22 | 0.09 | 0.169 |
| (1990 – 1994) $\times f_{1}(age)$ | -6.82 | 0.90 | 0.093 |
| (1990 – 1994) $\times f_{2}(age)$ | 3.80 | 0.30 | 0.455 |
| (1990 – 1994) $\times f_{3}(age)$ | 6.50 | 1.46 | 0.210 |
| (1995 – 1999) $\times f_{1}(age)$ | -10.44 | 0.29 | 0.001 |
| (1995 – 1999) $\times f_{2}(age)$ | 27.93 | 1.44 | <0.0001 |
| (1995 – 1999) $\times f_{3}(age)$ | 28.22 | 1.70 | <0.0001 |
| (> 1999) $\times f_{1}(age)$ | -2.64 | 0.20 | 0.713 |
| (> 1999) $\times f_{2}(age)$ | 11.25 | 0.21 | 0.089 |
| (> 1999) $\times f_{3}(age)$ | -7.18 | 0.29 | 0.397 |
| MRSA$\times f_{1}(age)$ | -2.36 | 0.47 | 0.489 |
| MRSA $\times f_{2}(age)$ | -0.67 | 0.26 | 0.845 |
| MRSA $\times f_{3}(age)$ | 0.11 | 0.80 | 0.975 |
| Pa$\times f_{1}(age)$ | -1.42 | 1.01 | 0.583 |
| Pa $\times f_{2}(age)$ | -9.78 | 0.61 | <0.0001 |
| Pa $\times f_{3}(age)$ | -5.68 | 0.64 | 0.020 |
| CFRD$\times f_{1}(age)$ | -5.26 | 0.62 | 0.233 |
| CFRD $\times f_{2}(age)$ | -10.32 | 1.15 | 0.054 |
| CFRD $\times f_{3}(age)$ | -6.25 | 0.11 | 0.127 |
| $On pancreatic enzymes$ $\times f_{1}(age)$ | -3.49 | 0.50 | 0.133 |
| On pancreatic enzymes $\times f_{2}(age)$ | -4.91 | 1.52 | 0.199 |
| On pancreatic enzymes $\times f_{3}(age)$ | 2.98 | 1.10 | 0.387 |
| $\# of visits per year (rolling)$ $\times f_{1}(age)$ | 4.84 | 0.42 | 0.465 |
| # of visits per year (rolling)$\times f_{2}(age)$ | 1.39 | 0.53 | 0.835 |
| # of visits per year (rolling)$\times f_{3}(age)$ | -3.52 | 0.49 | 0.618 |

Survival Submodel:

|  | Parameter Estimate | SE | P-value |
| --- | --- | --- | --- |
| Low SES | 0.32 | 0.01 | <0.0001 |
| Male | -0.20 | 0.01 | 0.001 |
| On pancreatic enzymes | -0.17 | 0.00 | 0.013 |
| Pa | -0.32 | 0.01 | <0.0001 |
| MRSA | 0.26 | 0.01 | <0.0001 |
| CFRD | -0.29 | 0.01 | 0.003 |

Survival (association parameters):

|  | Alpha coefficient | SE | P-value |
| --- | --- | --- | --- |
| FEV1 value | -0.0334 | 7e-04 | 0.003 |
| WFA value | 0.0006 | 2e-04 | 0.761 |

**Table S5: Joint Model with Longitudinal FEV1, WFA, HFA and PE onset**

FEV1 Submodel:

|  | Parameter Estimate | SE | P-value |
| --- | --- | --- | --- |
| Intercept | 76.49 | 0.20 | <0.0001 |
| Age-related spline terms |  |  |  |
| $f_{1}(age)$ | 3.87 | 0.51 | 0.568 |
| $f_{2}(age)$ | -13.91 | 0.59 | 0.037 |
| Genotype |  |  |  |
| Heterozygous | 1.78 | 0.23 | 0.061 |
| None/Unknown | 1.13 | 0.42 | 0.440 |
| Ref: Homozygous |  |  |  |
| Male | -0.68 | 0.03 | 0.334 |
| Hispanic Ethnicity | 6.70 | 0.53 | <0.0001 |
| Low SES | -0.36 | 0.01 | 0.021 |
| Birth Cohort |  |  |  |
| 1981 – 1989 | 2.97 | 0.14 | 0.255 |
| 1990 – 1994 | 6.44 | 0.30 | 0.015 |
| 1995 – 1999 | 9.45 | 0.14 | <0.0001 |
| > 1999 | 11.73 | 0.17 | <0.0001 |
| Ref: < 1981 |  |  |  |
| MRSA | -0.81 | 0.01 | <0.0001 |
| Pa | -0.33 | 0.08 | 0.142 |
| CFRD | -1.28 | 0.16 | 0.001 |
| On pancreatic enzymes | 0.58 | 0.09 | <0.0001 |
| # of visits per year (rolling) | 0.01 | 0.01 | 0.754 |
| Heterozygous $\times f_{1}(age)$ | 0.73 | 1.11 | 0.789 |
| Heterozygous $\times f_{2}(age)$ | 1.84 | 1.27 | 0.532 |
| None/Unknown $\times f_{1}(age)$ | 4.77 | 1.02 | 0.113 |
| None/Unknown $\times f_{2}(age)$ | 6.98 | 0.17 | <0.0001 |
| Male $\times f_{1}(age)$ | 5.45 | 0.13 | 0.001 |
| Male $\times f_{2}(age)$ | 2.37 | 0.12 | 0.071 |
| Hispanic Ethnicity $\times f_{1}(age)$ | -1.90 | 0.49 | 0.579 |
| Hispanic Ethnicity $\times f_{2}(age)$ | 0.52 | 1.11 | 0.905 |
| Low SES $\times f_{1}(age)$ | -9.21 | 0.14 | <0.0001 |
| Low SES$\times f_{2}(age)$ | -3.79 | 0.24 | 0.004 |
| (1981 – 1989) $\times f_{1}(age)$ | -4.69 | 0.22 | 0.300 |
| (1981 – 1989) $\times f_{2}(age)$ | -0.32 | 0.13 | 0.861 |
| (1990 – 1994) $\times f_{1}(age)$ | -7.31 | 0.26 | 0.103 |
| (1990 – 1994) $\times f_{2}(age)$ | 0.25 | 0.48 | 0.928 |
| (1995 – 1999) $\times f_{1}(age)$ | -6.22 | 0.23 | 0.149 |
| (1995 – 1999) $\times f_{2}(age)$ | 1.84 | 1.06 | 0.647 |
| (> 1999) $\times f_{1}(age)$ | -4.92 | 0.56 | 0.453 |
| (> 1999) $\times f_{2}(age)$ | 2.01 | 0.54 | 0.828 |
| MRSA$\times f_{1}(age)$ | -8.42 | 1.16 | 0.003 |
| MRSA $\times f_{2}(age)$ | -4.38 | 1.59 | 0.284 |
| Pa$\times f_{1}(age)$ | -8.52 | 0.79 | <0.0001 |
| Pa $\times f_{2}(age)$ | -5.53 | 0.75 | 0.003 |
| CFRD$\times f_{1}(age)$ | -10.39 | 0.93 | 0.003 |
| CFRD $\times f_{2}(age)$ | -5.14 | 0.78 | 0.123 |
| $On pancreatic enzymes$ $\times f_{1}(age)$ | -0.90 | 0.19 | 0.579 |
| On pancreatic enzymes $\times f_{2}(age)$ | 0.45 | 0.08 | 0.779 |
| $\# of visits per year (rolling)$ $\times f_{1}(age)$ | 0.88 | 0.32 | 0.881 |
| # of visits per year (rolling)$\times f_{2}(age)$ | -5.87 | 0.54 | 0.318 |

WFA Submodel:

|  | Parameter Estimate | SE | P-value |
| --- | --- | --- | --- |
| Intercept | 26.17 | 0.18 | <0.0001 |
| Age-related spline terms |  |  |  |
| $f_{1}(age)$ | 7.73 | 0.44 | 0.269 |
| $f_{2}(age)$ | -3.82 | 0.36 | 0.556 |
| Genotype |  |  |  |
| Heterozygous | 4.01 | 0.39 | 0.015 |
| None/Unknown | 3.68 | 0.57 | 0.114 |
| Ref: Homozygous |  |  |  |
| Male | 3.98 | 0.35 | 0.004 |
| Hispanic Ethnicity | 1.68 | 0.47 | 0.527 |
| Low SES | 0.08 | 0.18 | 0.719 |
| Birth Cohort |  |  |  |
| 1981 – 1989 | -10.70 | 0.14 | <0.0001 |
| 1990 – 1994 | 6.55 | 0.16 | 0.021 |
| 1995 – 1999 | 11.10 | 0.15 | <0.0001 |
| > 1999 | 14.69 | 0.13 | <0.0001 |
| Ref: < 1981 |  |  |  |
| MRSA | 0.18 | 0.25 | 0.827 |
| Pa | 0.51 | 0.09 | 0.007 |
| CFRD | -0.08 | 0.01 | 0.761 |
| On pancreatic enzymes | 0.16 | 0.01 | 0.082 |
| # of visits per year (rolling) | 0.19 | 0.00 | <0.0001 |
| Heterozygous $\times f_{1}(age)$ | 2.85 | 1.10 | 0.467 |
| Heterozygous $\times f_{2}(age)$ | 1.86 | 2.21 | 0.669 |
| None/Unknown $\times f_{1}(age)$ | 7.33 | 1.09 | 0.081 |
| None/Unknown $\times f_{2}(age)$ | 4.19 | 0.30 | 0.161 |
| Male $\times f_{1}(age)$ | -11.09 | 1.28 | <0.0001 |
| Male $\times f_{2}(age)$ | -12.81 | 0.63 | <0.0001 |
| Hispanic Ethnicity $\times f_{1}(age)$ | 4.90 | 1.89 | 0.471 |
| Hispanic Ethnicity $\times f_{2}(age)$ | 8.44 | 0.40 | 0.037 |
| Low SES $\times f_{1}(age)$ | -6.64 | 0.08 | <0.0001 |
| Low SES$\times f_{2}(age)$ | -4.37 | 0.36 | 0.055 |
| (1981 – 1989) $\times f_{1}(age)$ | 12.57 | 0.53 | 0.029 |
| (1981 – 1989) $\times f_{2}(age)$ | 11.82 | 0.46 | <0.0001 |
| (1990 – 1994) $\times f_{1}(age)$ | -14.92 | 0.25 | 0.003 |
| (1990 – 1994) $\times f_{2}(age)$ | 11.12 | 0.56 | 0.001 |
| (1995 – 1999) $\times f_{1}(age)$ | -6.69 | 0.24 | 0.157 |
| (1995 – 1999) $\times f_{2}(age)$ | 16.13 | 0.65 | <0.0001 |
| (> 1999) $\times f_{1}(age)$ | -5.86 | 0.25 | 0.371 |
| (> 1999) $\times f_{2}(age)$ | 1.83 | 0.32 | 0.861 |
| MRSA$\times f_{1}(age)$ | -3.64 | 0.06 | 0.245 |
| MRSA $\times f_{2}(age)$ | -2.04 | 1.51 | 0.713 |
| Pa$\times f_{1}(age)$ | -8.18 | 0.99 | 0.001 |
| Pa $\times f_{2}(age)$ | -5.59 | 0.31 | 0.021 |
| CFRD$\times f_{1}(age)$ | -9.55 | 1.35 | 0.029 |
| CFRD $\times f_{2}(age)$ | -6.40 | 0.12 | 0.111 |
| $On pancreatic enzymes$ $\times f_{1}(age)$ | -7.54 | 1.46 | 0.013 |
| On pancreatic enzymes $\times f_{2}(age)$ | 1.74 | 1.01 | 0.616 |
| $\# of visits per year (rolling)$ $\times f_{1}(age)$ | 10.32 | 0.35 | 0.097 |
| # of visits per year (rolling)$\times f_{2}(age)$ | -2.79 | 0.43 | 0.695 |

HFA Submodel:

|  | Parameter Estimate | SE | P-value |
| --- | --- | --- | --- |
| Intercept | 16.16 | 0.20 | <0.0001 |
| Age-related spline terms |  |  |  |
| $f_{1}(age)$ | 7.90 | 0.40 | 0.221 |
| $f_{2}(age)$ | 4.68 | 0.33 | 0.485 |
| Genotype |  |  |  |
| Heterozygous | 4.26 | 0.66 | 0.029 |
| None/Unknown | 3.83 | 1.48 | 0.265 |
| Ref: Homozygous |  |  |  |
| Male | 2.11 | 0.37 | 0.171 |
| Hispanic Ethnicity | 5.48 | 0.10 | 0.006 |
| Low SES | -0.08 | 0.06 | 0.645 |
| Birth Cohort |  |  |  |
| 1981 – 1989 | 2.56 | 0.55 | 0.439 |
| 1990 – 1994 | 6.20 | 0.17 | 0.029 |
| 1995 – 1999 | 10.99 | 0.24 | <0.0001 |
| > 1999 | 17.34 | 0.52 | <0.0001 |
| Ref: < 1981 |  |  |  |
| MRSA | -0.14 | 0.14 | 0.628 |
| Pa | 0.14 | 0.09 | 0.573 |
| CFRD | 0.12 | 0.24 | 0.969 |
| On pancreatic enzymes | 0.07 | 0.01 | 0.215 |
| # of visits per year (rolling) | -0.13 | 0.01 | <0.0001 |
| Heterozygous $\times f_{1}(age)$ | -0.72 | 1.50 | 0.823 |
| Heterozygous $\times f_{2}(age)$ | -0.46 | 0.64 | 0.890 |
| None/Unknown $\times f_{1}(age)$ | 0.68 | 1.84 | 0.968 |
| None/Unknown $\times f_{2}(age)$ | -2.27 | 0.40 | 0.486 |
| Male $\times f_{1}(age)$ | -2.77 | 0.82 | 0.367 |
| Male $\times f_{2}(age)$ | 1.80 | 0.35 | 0.433 |
| Hispanic Ethnicity $\times f_{1}(age)$ | 5.48 | 1.24 | 0.277 |
| Hispanic Ethnicity $\times f_{2}(age)$ | 7.92 | 1.27 | 0.106 |
| Low SES $\times f_{1}(age)$ | -8.67 | 0.74 | <0.0001 |
| Low SES$\times f_{2}(age)$ | -3.48 | 0.35 | 0.125 |
| (1981 – 1989) $\times f_{1}(age)$ | -4.22 | 1.21 | 0.485 |
| (1981 – 1989) $\times f_{2}(age)$ | -5.28 | 0.07 | 0.051 |
| (1990 – 1994) $\times f_{1}(age)$ | -6.91 | 0.56 | 0.187 |
| (1990 – 1994) $\times f_{2}(age)$ | -8.86 | 1.08 | 0.035 |
| (1995 – 1999) $\times f_{1}(age)$ | -2.82 | 1.25 | 0.645 |
| (1995 – 1999) $\times f_{2}(age)$ | -10.78 | 0.58 | 0.001 |
| (> 1999) $\times f_{1}(age)$ | 3.23 | 0.43 | 0.621 |
| (> 1999) $\times f_{2}(age)$ | 17.81 | 0.37 | 0.036 |
| MRSA$\times f_{1}(age)$ | -1.87 | 0.55 | 0.576 |
| MRSA $\times f_{2}(age)$ | 1.59 | 0.09 | 0.693 |
| Pa$\times f_{1}(age)$ | -3.09 | 0.50 | 0.175 |
| Pa $\times f_{2}(age)$ | -2.62 | 0.24 | 0.301 |
| CFRD$\times f_{1}(age)$ | -13.68 | 1.02 | 0.001 |
| CFRD $\times f_{2}(age)$ | -3.60 | 0.16 | 0.397 |
| $On pancreatic enzymes$ $\times f_{1}(age)$ | -2.83 | 0.90 | 0.331 |
| On pancreatic enzymes $\times f_{2}(age)$ | 4.67 | 1.56 | 0.213 |
| $\# of visits per year (rolling)$ $\times f_{1}(age)$ | 9.32 | 0.36 | 0.124 |
| # of visits per year (rolling)$\times f_{2}(age)$ | 0.21 | 0.49 | 0.990 |

Survival Submodel:

|  | Parameter Estimate | SE | P-value |
| --- | --- | --- | --- |
| SESlow1 | 0.33 | 0.01 | <0.0001 |
| Gender1 | -0.21 | 0.01 | 0.001 |
| isOnEnzymes1 | -0.19 | 0.00 | 0.007 |
| pa1 | -0.32 | 0.01 | <0.0001 |
| MRSA1 | 0.27 | 0.00 | <0.0001 |
| cfrd1 | -0.29 | 0.02 | 0.009 |

Survival (association parameters):

|  | Alpha coefficient | SE | P-value |
| --- | --- | --- | --- |
| FEV1 value | -0.0327 | 0.0006 | 0.005 |
| WFA value | 0.0022 | 0.0009 | 0.683 |
| HFA value | -0.0019 | 0.0011 | 0.737 |
