# Supplementary material for: Multivariate joint modeling to identify markers of growth and lung function decline that predict cystic fibrosis pulmonary exacerbation onset
Source: BMC Pulm Med. 2020 May 19;20:142. doi: 10.1186/s12890-020-1177-z (PMC7236487; doi:10.1186/s12890-020-1177-z)
Supplement: Supplementary file 2 — Additional file 2. Multivariate joint modeling to identify markers of growth and lung function decline that predict cystic fibrosis pulmonary exacerbation onset. [file 12890_2020_1177_MOESM2_ESM.pdf]

```
####################################################################
# Additional File 2                                                #
# Supplemental Material                                            #
# Multivariate joint modeling to identify markers of growth and    #
# lung function decline that predict cystic fibrosis pulmonary     #
# exacerbation onset                                               #
# Andrinopoulou, E.R., Clancy, J.P., & Szczesniak, R.D.            #
####################################################################

#################
# load packages #
#################

library(JMbayes)

############
# data set #
############

## The data set is called data

## Order the data set by id and time

### eDWID: id number
data <- data[order(data$age, data$age ), ]


## Data set for the Cox model (last measurement per patient)

data.id <- data[tapply(row.names(data), data$eDWID, tail, 1), ]


## Split data set in 3 parts -  ID is the identity number of each patient

## Same random split every time
set.seed(122017)

V <- 3 # split the data in 3 parts
n <- nrow(data.id)

splits <- split(data.id$ID, sample(rep(seq_len(V), length.out = n)))

data1 <- data[data$ID %in% splits$`1`, ]
data1.id <- data1[tapply(row.names(data1), data1$ID, tail, 1), ]

data2 <- data[data$ID %in% splits$`2`, ]
data2.id <- data2[tapply(row.names(data2), data2$ID, tail, 1), ]

data3 <- data[data$ID %in% splits$`3`, ]
data3.id <- data3[tapply(row.names(data3), data3$ID, tail, 1), ]

##########
# Models #
##########

### eDWID: id number
### fev outcome: FEV1
### bmipct outcome: BMI percentile
### age covariate
### F508_c covariate: Delta F508 gene (Homozygous/Heterozygous/Neither)
```

```
### Gender covariate
### HispanicRace covariate: yes/no
### SESlow covariate: Using state/federal or having no insurance is a
###                   marker of low socioeconomic status (Yes/No)
### bcohr covariate: Birth cohort for year patient was born, used to
###                  adjust for changes in standards of care and possible
###                  left truncation (1981-1988/1988-1994/After 1994)
### MRSA covariate: Methicillin-resistant Staphylococcus aureus (Yes/No)
### pa covariate: Pseudomonas aeruginosa (Yes/No)
### cfrd covariate: CF-related diabetes (Normal/Impaired/CFRD with
###                 or without fasting hyperglycemiaons)
### isOnEnzymes covariate: Taking a pancreatic enzyme supplement, marks
###                        pancreatic insufficiency (Yes/No)
### numVisityr covariate: Rolling total of number of visits within the
###                       year. Used to account for irregular
###                       follow up between patients
### pe1 survival outcome: occurrence of first recorded pulmonary
###                       exacerbation (Occurred/Not occurred)

## 1st subset

### Multivariate mixed-effects model

MixedModelFit1 <- mvglmer(list(fev ~ ns(age,3)*F508_c +
                                 ns(age,3)*Gender +
                                 ns(age,3)*HispanicRace +
                                 ns(age,3)*SESlow +
                                 ns(age,3)*bcohr + ns(age,3)*MRSA +
                                 ns(age,3)*pa  + ns(age,3)*cfrd +
                                 ns(age,3)*isOnEnzymes +
                                 ns(age,3)*numVisityr +
                                 (ns(age,3) | eDWID),
                               bmipct ~ ns(age,3)*F508_c +
                                 ns(age,3)*Gender +
                                 ns(age,3)*HispanicRace +
                                 ns(age,3)*SESlow +
                                 ns(age,3)*bcohr + ns(age,3)*MRSA +
                                 ns(age,3)*pa  + ns(age,3)*cfrd +
                                 ns(age,3)*isOnEnzymes +
                                 ns(age,3)*numVisityr +
                                 (ns(age,3) | eDWID)),  engine = c("JAGS"),
                          data = data1,
                          families = list(gaussian, gaussian))

### Cox model
CoxFit1 <- coxph(Surv(age, pe1) ~ SESlow + Gender + isOnEnzymes +
                   pa + MRSA + cfrd,
                 data = data1.id, model = TRUE)

### Joint model
JMFitBMI1 <- mvJointModelBayes(MixedModelFit1, CoxFit1, timeVar = "age")
summary(JMFitBMI1)

JM1 <- JMFitBMI1$mcmc

## 2nd subset

### Multivariate mixed-effects model

MixedModelFit2 <- mvglmer(list(fev ~ ns(age,3)*F508_c +
```

```
                                 ns(age,3)*Gender +
                                 ns(age,3)*HispanicRace +
                                 ns(age,3)*SESlow +
                                 ns(age,3)*bcohr + ns(age,3)*MRSA +
                                 ns(age,3)*pa  + ns(age,3)*cfrd +
                                 ns(age,3)*isOnEnzymes +
                                 ns(age,3)*numVisityr +
                                 (ns(age,3) | eDWID),
                               bmipct ~ ns(age,3)*F508_c +
                                 ns(age,3)*Gender +
                                 ns(age,3)*HispanicRace +
                                 ns(age,3)*SESlow +
                                 ns(age,3)*bcohr + ns(age,3)*MRSA +
                                 ns(age,3)*pa  + ns(age,3)*cfrd +
                                 ns(age,3)*isOnEnzymes +
                                 ns(age,3)*numVisityr +
                                 (ns(age,3) | eDWID)),  engine = c("JAGS"),
                          data = data2,
                          families = list(gaussian, gaussian))

### Cox model

CoxFit2 <- coxph(Surv(age, pe1) ~ SESlow + Gender + isOnEnzymes +
                   pa + MRSA + cfrd,
                 data = data2.id, model = TRUE)

### Joint model

JMFitBMI2 <- mvJointModelBayes(MixedModelFit2, CoxFit2, timeVar = "age")
summary(JMFitBMI2)

JM2 <- JMFitBMI2$mcmc

## 3rd subset

### Multivariate mixed-effects model

MixedModelFit3 <- mvglmer(list(fev ~ ns(age,3)*F508_c +
                                 ns(age,3)*Gender +
                                 ns(age,3)*HispanicRace +
                                 ns(age,3)*SESlow +
                                 ns(age,3)*bcohr + ns(age,3)*MRSA +
                                 ns(age,3)*pa  + ns(age,3)*cfrd +
                                 ns(age,3)*isOnEnzymes +
                                 ns(age,3)*numVisityr +
                                 (ns(age,3) | eDWID),
                               bmipct ~ns(age,3)*F508_c +
                                 ns(age,3)*Gender +
                                 ns(age,3)*HispanicRace +
                                 ns(age,3)*SESlow +
                                 ns(age,3)*bcohr + ns(age,3)*MRSA +
                                 ns(age,3)*pa  + ns(age,3)*cfrd +
                                 ns(age,3)*isOnEnzymes +
                                 ns(age,3)*numVisityr +
                                 (ns(age,3) | eDWID)),  engine = c("JAGS"),
                          data = data3,
                          families = list(gaussian, gaussian))

### Cox model
```

```
CoxFit3 <- coxph(Surv(age, pe1) ~ SESlow + Gender + isOnEnzymes +
                   pa + MRSA + cfrd,
                 data = data3.id, model = TRUE)

### Joint model

JMFitBMI3 <- mvJointModelBayes(MixedModelFit3, CoxFit3, timeVar = "age")
summary(JMFitBMI3)

JM3 <- JMFitBMI3$mcmc

#####################################
# Predictions for the univariate joint model #
####################################

## Fit a model

### Univariate mixed-effects model

MixedModelFit1 <- mvglmer(list(fev ~ ns(age,3)*F508_c +
                                 ns(age,3)*Gender +
                                 ns(age,3)*HispanicRace +
                                 ns(age,3)*SESlow +
                                 ns(age,3)*bcohr + ns(age,3)*MRSA +
                                 ns(age,3)*pa  + ns(age,3)*cfrd +
                                 ns(age,3)*isOnEnzymes +
                                 ns(age,3)*numVisityr +
                                 (ns(age,3) | eDWID)), engine = c("JAGS"),
                          data = data1,
                          families = list(gaussian))

### Cox model

CoxFit1 <- coxph(Surv(age, pe1) ~ SESlow + Gender + isOnEnzymes +
                   pa + MRSA + cfrd,
                 data = data1.id, model = TRUE)

### Joint model

JMFitBMI1 <- mvJointModelBayes(MixedModelFit1, CoxFit1, timeVar = "age")

## Select patient

newdata <- data[data$eDWID %in% unique(data$eDWID)[c(4)],]
newdata <- newdata[order(newdata$age), ]

## Obtain predictions

survPreds <- vector("list", nrow(newdata))
for (i in 1:nrow(newdata)) {
  survPreds[[i]] <- survfitJM(object = JMFitBMI1, newdata = newdata[1:i, ],
                              idVar = "eDWID")
}

## Obtain AUC

auc1 <- aucJM(JMFitBMI1, newdata = newdata, idVar = "eDWID",
              Tstart = 6, Thoriz = 6.5)
```

```
# Cross-validation procedure

CrossValJM <- function(j){
  # load packages
  library("JMbayes")
  library("splines")

  # training data set
  trainingData <- data[!data$eDWID %in% j, ]
  # training data set: last observation per patient
  trainingData.id <- trainingData[!duplicated(trainingData$eDWID), ]
  # testing dataset for prediction
  testingData <- data[data$eDWID %in% j, ]

  # fit mixed model on training data set
  MixedModelFit <- mvglmer(list(fev ~ ns(age,3)*F508_c + ns(age,3)*Gender +
                                ns(age,3)*HispanicRace +
                                ns(age,3)*SESlow +
                                ns(age,3)*bcohr + ns(age,3)*MRSA +
                                ns(age,3)*pa  + ns(age,3)*cfrd +
                                ns(age,3)*isOnEnzymes +
                                ns(age,3)*numVisityr +
                                (ns(age,3) | eDWID)), engine = c("JAGS"),
                           data = trainingData,
                           families = list(gaussian))

  # fit cox model on training data set
  CoxFit <- coxph(Surv(age + 0.1, pe1) ~ SESlow + Gender + isOnEnzymes +
                    pa + MRSA + cfrd,
                  data = trainingData.id, model = TRUE)

  # fit joint model on training data set
  JMFitBMI <- mvJointModelBayes(MixedModelFit, CoxFit, timeVar = "age")

  # obtain auc on testing data set
  auc1 <- aucJM(JMFitBMI, newdata = testingData, idVar = "eDWID",
              Tstart = 6, Thoriz = 6.5)
  auc2 <- aucJM(JMFitBMI, newdata = testingData, idVar = "eDWID",
              Tstart = 6, Thoriz = 7)
  auc3 <- aucJM(JMFitBMI, newdata = testingData, idVar = "eDWID",
              Tstart = 6, Thoriz = 8)
  auc4 <- aucJM(JMFitBMI, newdata = testingData, idVar = "eDWID",
              Tstart = 12, Thoriz = 12.5)
  auc5 <- aucJM(JMFitBMI, newdata = testingData, idVar = "eDWID",
              Tstart = 12, Thoriz = 13)
  auc6 <- aucJM(JMFitBMI, newdata = testingData, idVar = "eDWID",
              Tstart = 12, Thoriz = 14)
  auc7 <- aucJM(JMFitBMI, newdata = testingData, idVar = "eDWID",
              Tstart = 16, Thoriz = 16.5)
  auc8 <- aucJM(JMFitBMI, newdata = testingData, idVar = "eDWID",
              Tstart = 16, Thoriz = 17)
  auc9 <- aucJM(JMFitBMI, newdata = testingData, idVar = "eDWID",
              Tstart = 16, Thoriz = 18)

  # rerge results in a list
  list(auc1 = auc1, auc2 = auc2, auc3 = auc3,
       auc4 = auc4, auc5 = auc5, auc6 = auc6,
       auc7 = auc7, auc8 = auc8, auc9 = auc9)
}
```
